# Socioeconomic inequalities in patient-reported outcome measures among total hip and knee arthroplasty patients: a comprehensive analysis of instruments and domains

**DOI:** 10.1186/s12939-025-02520-4

**Published:** 2025-05-23

**Authors:** Joshua M. Bonsel, Max Reijman, Erin M. Macri, Jan A. N. Verhaar, Liza N. van Steenbergen, Gouke J. Bonsel

**Affiliations:** 1 Department of Orthopaedics and Sports Medicine, Erasmus MC, Rotterdam, The Netherlands; 2 Dutch Arthroplasty Register (Landelijke Registratie Orthopedische Interventies), ‘s Hertogenbosch, The Netherlands; 3 EuroQol Research Foundation, Rotterdam, The Netherlands

**Keywords:** Total hip arthroplasty, Total knee arthroplasty, Socioeconomic status, Inequality, Patient-reported outcome, Reporting heterogeneity

## Abstract

**Background:**

Prior to total hip and knee arthroplasty (THA/TKA), patients with low socioeconomic status (SES) report worse Patient-Reported Outcome Measures (PROMs), persisting postoperatively. This study explores which self-reported PROMs and their specific domains are most involved.

**Methods:**

We obtained data from the Dutch Arthroplasty Registry (2014–2022), including over 100,000 THA/TKA patients with complete preoperative and 12-month follow-up PROMs. The EQ-5D-3L and EQ-5D-5L, EQ Visual Analogue Scale (VAS), Oxford Hip and Oxford Knee Score (OHS/OKS), and Numerical Rating Scales (NRS) for pain and satisfaction (TKA only, at 12-month follow-up) were obtained. The PROMs were transformed to a 0-100 scale for direct comparison. A SES-indicator based on neighborhood income, unemployment rate, and education level was divided into quintiles, which are equal groups representing 20% of the Dutch population, and was ranked from least to most deprived. Through linear regression we contrasted the most and least deprived groups, adjusting for patient and surgical characteristics. The contribution (percentage) of each domain to the overall health inequalities was estimated for the EQ-5D’s and the OHS/OKS.

**Results:**

In TKA patients, the most compared to the least deprived group had a lower preoperative EQ-5D-3L score: -2.1 [95% confidence interval − 2.6, -1.6], *p* < 0.001. At 12-month follow-up, the difference was smaller: EQ-5D-3L 1.3 [-1.9, -0.7], *p* < 0.001. A larger difference between the most and least deprived was present in OKS (preoperatively: -4.3 [-5.3, -3.4], *p* < 0.001; 12-month: -1.8 [-2.5, -1.2], *p* < 0.001). The difference in EQ VAS was smaller (preoperatively: -0.8 [-1.5, -0.1], *p* = 0.024; 12-month: -0.5 [-1.2, 0.1], *p* = 0.108). The difference in NRS pain (in rest) was comparable to those in EQ-5D-3L and OKS (preoperatively: -4.5 [-5.4, -3.5], *p* < 0.001; 12-month: -2.7 [-3.5, -1.9], *p* < 0.001), while no difference between the most and least deprived in NRS satisfaction was observed at 12 months. For EQ-5D-3L, the domain usual activities accounted for up to 46% of the difference between SES groups, while anxiety/depression played a limited role (up to 17%). For OHS/OKS, functioning contributed most in THA (up to 61%) and pain contributed most in TKA (up to 68%). Differences in PROM scores between SES groups, and how these differences compared across PROMs, were similar in THA patients. Overall, the EQ-5D-5L produced similar patterns compared to the EQ-5D-3L.

**Conclusions:**

Deprived THA/TKA patients have poorer pre- and postoperative health, which was primarily related to worse functioning and pain; the clinical relevance of these differences remain uncertain. These differences did not translate into worse overall health (EQ VAS) or into higher dissatisfaction among deprived patients. Future research should investigate whether the EQ VAS and satisfaction measure reflected health differences between SES groups or were biased by reporting heterogeneity, where respondents interpreted the wording differently.

**Supplementary Information:**

The online version contains supplementary material available at 10.1186/s12939-025-02520-4.

## Background

Socioeconomic status (SES), which is often defined by factors such as education, income, and occupation, is a significant driver of health inequalities in most medical fields [1, 2]. The impact of SES on health outcomes is also evident in orthopedic procedures such as total hip and knee arthroplasty (THA/TKA). Total hip and knee arthroplasty are two commonly performed procedures that typically alleviate pain and symptoms in patients suffering from end-stage osteoarthritis of the hip and knee [3]. The most deprived THA/TKA patients (i.e., those with lower SES) experience poorer clinical outcomes such as increased mortality and complication rates [4]. The health disadvantage associated with low SES may arise from several mechanisms, including increased risk exposure, less healthy occupational environment, and reduced access to medical care.

Patient-Reported Outcome Measures (PROMs) provide a valuable alternative to abovementioned clinical metrics for measuring health inequalities. Patient-Reported Outcome Measures are standardized, validated, mostly self-reported questionnaires that assess patients’ generic and condition-specific health status, and are widely used in orthopedics, including THA/TKA. For example, previous studies have found that low SES associates with poorer preoperative [5–10] and postoperative PROMs [6, 8–15], which have illuminated that inequalities occur in the starting position before surgery and during recovery after THA/TKA. The cited studies employed various PROMs to explore health differences by SES, and reported varying magnitudes of differences across PROMs. Besides differing patient populations, multiple instrument-specific factors and design features may be responsible, hampering our understanding of how inequalities manifest across these PROM instruments. The factors/features are described below:

First, the instrument construct, including the health domains covered, matters [16]. For example, the Oxford Hip and Knee Score (OHS/OKS) instruments assess similar constructs, with emphasis on joint-related function and pain. In comparison, the EQ-5D covers five generic quality of life domains: mobility, self-care, usual activities, pain/discomfort, and anxiety/depression. As it turns out, the OHS and EQ-5D provide similar results in THA patients, with the exception of anxiety/depression, a domain with independent relevance [17]. In our context, this construct difference might impact measured health differences by SES, if deprived patients disproportionately experience mental issues following surgery.

Other differences in instrument characteristics, such as the scale used, may also matter, as they can affect validity, reliability and responsiveness [16]. For example, instrument-specific factors like double-barreled items which combine multiple concepts into a single question or varying response formats may contribute to differences in measurement properties. Generally, multi-item measures such as the OHS/OKS and EQ-5D psychometrically perform better regarding the aforementioned measurement properties compared to single-item measures such as the EQ Visual Analogue Scale (VAS) and Numerical Rating Scale (NRS) for pain in THA and TKA patients [18–21]. Consequently, single-item measures such as the EQ VAS and Numerical Rating Scale (NRS) for pain may show a less pronounced negative effect of SES on health compared to the multi-item OHS/OKS and EQ-5D.

A third factor potentially affecting the size of SES-related health differences is reporting heterogeneity, which refers to the phenomenon where a respondent systematically interprets questions or response options differently. A simple example is the avoidance of extreme answers, more often observed in the aged [22]. In our context, it is known that poor individuals express greater satisfaction with healthcare than their more affluent counterparts, even when they show inferior outcomes measured by other metrics [23]. Reporting heterogeneity may therefore induce underestimation of ‘true’ health differences by SES. While all PROMs are susceptible to this phenomenon, some may be more susceptible than others, such as the EQ VAS and satisfaction questions in general. We believe this to be the case due to the phrasing used in these instruments (i.e., “How would you rate your health today?” and “How satisfied are you with the treatment received?“, respectively). Such phrasing may invite patients to respond using their own reference of the quality of care received, more so compared to the OHS/OKS or EQ-5D. While accounting for reporting heterogeneity is complex, it should be considered as an explanans when comparing PROMs, for example in assessments of health inequality magnitude [24].

The purpose of this study is to explore SES-related health differences in THA and TKA patients using a range of PROMs and to examine how the magnitude of these differences relates to their domain structure and scale type. By using data from a single-nation arthroplasty registry, potential variations due to differences in patient populations are minimized. Our study departs from the assumption that, among all types of measures, health measured using the EQ-5D and OHS/OKS best reflect ‘true’ health in the studied population. We propose the following hypotheses:

### Hypothesis 1

The negative impact of SES will be more pronounced when measured by the EQ-5D compared to the OHS/OKS, because the EQ-5D includes the domain of anxiety/depression.

### Hypothesis 2

The negative impact of SES will be more pronounced in multi-item scales (e.g., EQ-5D or OHS/OKS) compared to single-item scales (e.g., EQ VAS, NRS pain and satisfaction), due to the poorer instrument characteristics of single-item scores in general.

### Hypothesis 3

The negative impact of SES will be most pronounced when measured with the EQ-5D and OHS/OKS compared to the EQ VAS and NRS satisfaction, which may suggest the presence of reporting heterogeneity.

## Methods

### Data source

This observational cohort study used anonymized, prospectively collected clinical data from the Dutch Arthroplasty Registry (LROI; www.lroi.nl). This registry is under responsibility of the Netherlands Orthopedic Association (NOV). Patients undergoing surgery may opt out for sharing their data with the LROI. Studies using LROI data are subject to technical and ethical judgment by the registry holder, and Dutch Law does not require additional institutional ethical judgment by a Medical Ethical Review Board. Our study followed the STROBE guideline for observational studies and when appropriate the COSMIN Study Design checklist [25, 26].

The LROI captures over 95% of THA and TKA surgeries performed in the Netherlands since 2009 [27]. Variables encompass patient and surgical characteristics, and outcomes. Since 2014, a set of internationally validated generic and disease-specific PROMs has been included in the LROI, supported by the NOV. PROMs are collected at three time points: preoperatively (maximal 6 months before surgery), 3 months after surgery (range 2–4 months) in THA, 6 months after surgery (range 5–7 months) in TKA, and 12-months after surgery (range 11–13 months) for both procedures. Response rates since 2017 are approximately 40%, depending largely on hospital participation [28].

### Inclusion criteria

We selected primary THA and TKA patients between 2014 and 2022 with complete preoperative and 12-month follow-up PROMs. Further selection was through diagnosis of osteoarthritis, which is the largest and most homogeneous group. Records of contralateral joint replacements during this period were also obtained. From 2014 until 2020, the EQ-5D-3L was used. In 2021, it was replaced by the EQ-5D-5L. Since scores of the − 3L and − 5L version are not directly interchangeable on the individual level (see ‘Outcomes: PROMs’ below) [29], the dataset was split accordingly. Patients who completed a preoperative EQ-5D-3L and responded to a 12-month follow-up EQ-5D-5L during the transition period were excluded.

### Variables

Patient and surgical data included age, biological sex, body mass index (BMI), Charnley score [30], American Society of Anesthesiologists (ASA) score [31], previous surgery of the replaced joint, smoking status, type of hospital, prothesis fixation method, and surgical approach. The Charnley score represents the extent of osteoarthritis disease, and ranges from “A” (one joint affected) to “C” (multiple joints affected or quality of life severely impaired due to the disease). Hospital type was categorized as private, general, or university. Fixation methods were categorized as cemented, cementless, or hybrid. Surgical approach for THA was categorized as anterior, anterolateral, posterolateral, straight lateral, and other. For TKA, surgical approach was categorized as lateral parapatellar, medial parapatellar, and mid- or sub-vastus.

### Exposure: socioeconomic status

A neighborhood SES score was linked to patients using four-digit postal codes [32]. This standardized score is calculated by two government institutions (Statistics Netherlands and the Netherlands Institute for Social Research) from the mean income per household, percentage of households with low income, percentage of unemployed inhabitants, and percentage of households with low education per postal code area. The SES score is only calculated for postal code areas with a minimum of 100 inhabitants (mean 4300 inhabitants per postal code). We used the 2017 scoring because it was the mid-point of our primary cohort. Moreover, neighborhood deprivation is known to be very stable over several years [33]. Based on customary practice and guidelines the SES score was divided into quintiles, which are equal groups representing 20% of the Dutch population, ranked from least deprived (Q1) to most deprived group (Q5) [34].

### Outcomes: proms

For our research questions, we obtained data from EQ-5D-3L, EQ-5D-5L, EQ VAS, OHS/OKS, NRS for pain at rest, NRS for pain during activity, and NRS for satisfaction with the undergone procedure. All PROMs were self-reported.

#### EQ-5D

The EQ-5D-3L and EQ-5D-5L have 5 domains (mobility, self-care, usual activities, pain/discomfort, and anxiety/depression) on which patients report their perceived general health [35]. For each domain the − 3L version has 3 response options ranging from ‘extreme’ to ‘no complaints’. The EQ-5D-5L has 5 response options, which has been shown to increase sensitivity and reducing ceiling [29]. Typically, the scores for the 5 domains can be linked to a ‘value set’, which transforms the 5 domain scores into an overall ‘utility’ value for this health state. When the purpose of EQ-5D data is non-economic, as in this study, the EuroQol Research Foundation advises to sum the domain scores directly into a level-sum-score (LSS) [36]. The LSS of the EQ-5D-3L ranges from 5 to 15, and for the EQ-5D-5L it ranges from 5 to 25, with lower scores indicating better health.

#### EQ VAS

The EQ VAS rates the respondent’s health on a single visual analogue scale from 0 (‘The worst health you can imagine’) to 100 (‘The best health you can imagine’).

#### OHS/OKS

The OHS/OKS consists of 12 items on two domains, namely (physical) functioning and pain, in patients with osteoarthritis of the hip/knee [37]. In the OHS each domain is covered with 6 items, while in the OKS the function and pain domains are covered by 5 and 7 items, respectively [38, 39]. The OHS/OKS scores range from 0 to 48, with 48 indicating no disability.

#### NRS pain

The NRS pain outcomes are rated on a scale of 0 to 10, with 10 reflecting severe pain.

#### NRS satisfaction

The NRS satisfaction score was only available for patients in the TKA cohort at 12-month follow-up. For the NRS satisfaction, however, a score of 10 reflects the highest degree of satisfaction with the result.

### Missing data

Missing Charnley scores were conservatively estimated as ‘A’, because only patients with osteoarthritis were selected. Ages under 10 years or over 105 years, and BMI values below 10 or above 70, were recoded as ‘missing’ in accordance with LROI guidelines [40]. As missing data in one or more variables was present in only 4% of patients with EQ-5D data, a complete case analysis was conducted. Among patients with EQ-5D data, other PROMs were missing in up to 9% of patients per cohort; therefore, a complete case analysis was also conducted for these outcomes.

### Statistical analysis

Analyses were performed for THA and TKA separately, and for the primary (EQ-5D-3L) and secondary (EQ-5D-5L) cohorts. For continuous variables we calculated medians with interquartile ranges (IQR), and for categorical variables percentages. To quantify ‘ceiling’ we calculated the proportion of patients reporting best health (i.e., a score of 100) for each total PROM score. Based on existing reporting practice, a percentage greater than 15% was considered indicative of a ceiling. The ANOVA (t-test) and chi-squared tests were used to compare the most and least deprived groups when appropriate.

First, we explored the size of SES-related health differences across different PROMs. To facilitate direct comparison, all PROM scores were transformed to a 0 to 100-scale, with 100 representing the best attainable outcome. Linear regression (LR) was used to test and quantify the association between SES groups and total PROMs scores, separately for the preoperative and 12-month follow-up measurement. The least deprived SES group was used as reference; we expected negative regression coefficients for the more deprived SES categories. Models were adjusted for sex, age, BMI, ASA score, Charnley score, and type of hospital, which were considered potential confounders [10]. The regression models for 12-month follow-up outcomes were also adjusted for the preoperative score of the respective PROM [41]. To facilitate meaningful interpretation of coefficients, the preoperative score was entered as a categorical variable, grouped into tertiles (low, medium, high) of approximately equal size using the ‘santoku’ package [42]. Cut-off values are included in Supplemental File 1, Table 1.

Previously, we found that the relation between SES and postoperative PROMs differed according to preoperative score [10]. Therefore, we also stratified the models according to the tertiles of the preoperative score of the respective PROM, rather than adjusting for the preoperative score. No preoperative measurement is available for the NRS satisfaction, which should be regarded as a concept both covering current health and health change. As satisfaction following arthroplasty is known to be associated with preoperative health we may expect stronger associations between SES and satisfaction for worse preoperative health [43]. Thus, NRS satisfaction was also stratified according to the preoperative EQ-5D LSS.

A three-step procedure was conducted to estimate the contribution of separate domains of the PROMs (EQ-5D and OHS/OKS) to the health differences by SES. First, we removed the domain for which the contribution was to be calculated from the total score of that PROM [44]. The model was re-run with this ‘total minus one’ score. Second, the SES coefficients of the total and ‘total minus one’ model were compared, and the difference in coefficients was calculated as a percentage. Third, the percentages over the SES groups were averaged, resulting in a percentage-wise expression of how much a domain contributed to the overall association between SES and the total score. We repeated this procedure for each domain of the PROM. For the OKS, if each domain contributed equally, the functional domain would account for around 42% (5/12 items) and the pain domain would account for 58% (7/12 items) of the health difference found with the total OKS score. The OHS and EQ-5D have a balanced number of items across their domains, so equal contributions would result in percentages of equal size.

Because our dataset contained contralateral procedures, we evaluated whether we needed to account for nesting of outcomes at the patient level (i.e., using hierarchical modeling). We compared empty (null) models with and without random intercepts for the patient. As model fit did not improve, we reported the results of regular regression models.

We compared coefficients between PROMs, presenting them with 95% confidence intervals [95% CIs] and p-values to determine the degree of certainty in differences in inequalities observed. We accepted/refuted our hypotheses based on whether the difference between coefficients exceeded the uncertainty interval (i.e., showed no overlap) in the expected direction, following standard practice for evaluating hypotheses in the validation of PROM instruments. In our previous publication, no evidence of bias was observed in the inequality patterns due to non-response; hence, this was not assessed again [10]. A p-value of < 0.05 was considered statistically significant. All analyses were performed in R version 4.1.2 [45].

## Results

**Fig. 1:**
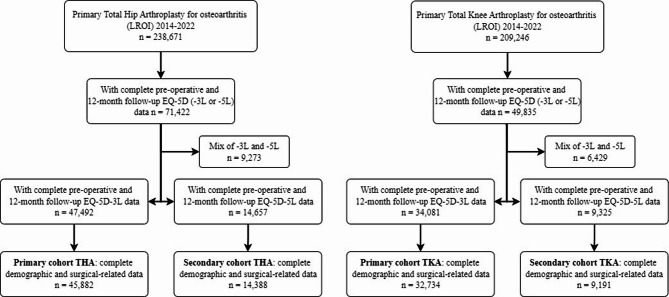
Flowchart depicting case-selection (THA/TKA)

After removing incomplete cases, the primary (EQ-5D-3L) cohorts had 45,822 THA and 32,734 TKA procedures (Fig. 1). The secondary (EQ-5D-5L) cohorts had 14,388 THA and 9,191 TKA procedures.

### Primary (EQ-5D-3L) cohorts

#### Descriptive analysis

Total Hip and Knee Arthroplasty patients were about 69 (IQR 63 to 75) years old and were more likely to be female (63%). Health differences by SES followed expected patterns. For example, those who were more deprived tended to be less healthy measured with ASA score (Supplemental File 1, Tables 2 and 3).

Preoperatively, median values for the OHS/OKS and NRS pain scores were lower than those for the EQ-5D-3L and EQ VAS scores, and were closer to the scales’ midrange score of 50 (i.e., the center of the 0-100 scale) for all SES groups. The OHS/OKS and NRS pain scores also showed greater distribution (larger IQRs). At 12-month follow-up, median values and distributions for all SES groups were similar across all PROMs.

Preoperatively, there was no ceiling in any of the PROMs across SES groups. At 12-month follow-up, all PROMs exhibited ceiling to some extent. The highest was observed for the NRS pain outcomes, while the lowest was observed for EQ VAS. The most deprived group responded best health about 5% less often for all PROMs.

### Linear regression models

The full adjusted models can be found in Supplemental File 1, Tables 4, 5, 6 and 7.

#### Hypothesis 1

*The negative impact of SES will be more pronounced when measured using the EQ-5D compared to the OHS/OKS*.

Preoperatively, the most deprived group had lower EQ-5D-L LSS (THA: -1.6 [95% CI -2.0, -1.2], *p* < 0.001; TKA: -2.1 [95% CI -2.6, -1.6], *p* < 0.001) scores than the least deprived group (Table 1). The differences on OHS/OKS compared to EQ-5D-3L scores was similar in THA, but larger in TKA patients with non-overlapping CIs (THA: -2.4 [95% CI -3.0, -1.9], *p* < 0.001); TKA: -4.3 [95% CI -5.3, -3.4], *p* < 0.001). At 12-month follow-up, differences were smaller (Table 2). The most deprived group reported lower EQ-5D-3L LSS (THA: -1.1 [95% CI -1.6, -0.7], *p* < 0.001; TKA: -1.3 [95% CI -1.9, -0.7], *p* < 0.001) and OHS/OKS (THA: -0.7 [95% CI -1.1,-0.2], *p* = 0.004; TKA: -1.8 [95% CI -2.5,-1.2], *p* < 0.001) scores. The results indicate that this hypothesis was *not met*.

**Table 1:** Association between socioeconomic status and preoperative health status

	EQ-5D-3L LSS		EQ VAS		OHS/OKS		NRS Pain in rest		NRS Pain during activity	
Variables	Beta [95% CI]	*p*-value	Beta [95% CI]	*p*-value	Beta [95% CI]	*p*-value	Beta [95% CI]	*p*-value	Beta [95% CI]	*p*-value
**Total Hip Arthroplasty**										
Intercept	61.5 [60.6, 62.4]	< 0.001	67.4 [66.2, 68.7]	< 0.001	50.5 [50.0, 50.9]	< 0.001	45.1 [43.4, 46.8]	< 0.001	25.5 [24.2, 26.9]	< 0.001
SES										
Q1 [least depr.]	ref		ref		ref		ref		ref	
Q2	-0.5 [-1.0, -0.1]	0.013	-0.8 [-1.4, -0.3]	0.005	-0.7 [-1.2, -0.1]	0.019	-1.0 [-1.8, -0.3]	0.008	-0.7 [-1.3, -0.1]	0.021
Q3	-1.1 [-1.5, -0.7]	< 0.001	-0.8 [-1.3, -0.2]	0.008	-1.4 [-1.9, -0.8]	< 0.001	-1.7 [-2.5, -1.0]	< 0.001	-1.2 [-1.8, -0.6]	< 0.001
Q4	-1.4 [-1.8, -1.0]	< 0.001	-0.6 [-1.1, 0.0]	0.048	-1.8 [-2.4, -1.3]	< 0.001	-2.4 [-3.1, -1.6]	< 0.001	-2.1 [-2.7, -1.5]	< 0.001
Q5 [most depr.]	-1.6 [-2.0, -1.2]	< 0.001	-1.0 [-1.6, -0.4]	0.001	-2.4 [-3.0, -1.9]	< 0.001	-3.4 [-4.2, -2.7]	< 0.001	-1.5 [-2.2, -0.9]	< 0.001
R-squared	0.05		0.04		0.08		0.03		0.03	
**Total Knee Arthroplasty**										
Intercept	65.4 [64.1, 66.8]		71.4 [69.4, 73.4]		51.2 [49.0, 53.3]		45.7 [43.0, 48.5]		26.0 [23.9, 28.2]	
SES										
Q1 [least depr.]	ref		ref		ref		ref		ref	
Q2	-0.5 [-1.0, 0.0]	0.046	0.6 [-0.1, 1.3]	0.111	-1.4 [-2.4, -0.5]	0.002	-0.8 [-1.8, 0.2]	0.112	-0.7 [-1.4, 0.1]	0.094
Q3	-1.2 [-1.7, -0.7]	< 0.001	0.3 [-0.4, 0.9]	0.441	-2.0 [-2.9, -1.1]	< 0.001	-2.5 [-3.5, -1.6]	< 0.001	-1.8 [-2.5, -1.0]	< 0.001
Q4	-1.5 [-2.0, -1.1]	< 0.001	0.2 [-0.5, 0.8]	0.646	-1.9 [-2.8, -1.0]	< 0.001	-3.4 [-4.3, -2.5]	< 0.001	-2.0 [-2.7, -1.3]	< 0.001
Q5 [most depr.]	-2.1 [-2.6, -1.6]	< 0.001	-0.8 [-1.5, -0.1]	0.024	-4.3 [-5.3, -3.4]	< 0.001	-4.5 [-5.4, -3.5]	< 0.001	-1.9 [-2.6, -1.2]	< 0.001
R-squared	0.05		0.05		0.09		0.03		0.03	

The table depicts data from the EQ-5D-3L cohort. Multivariable linear regression models were used. The LSS, OHS, OKS and NRS outcomes were transformed to 0-100 where 100 is the best score possible. The regression models were adjusted for sex, age, BMI, ASA score, Charnley score, and type of hospital

LSS = level sum score

VAS = Visual Analogue Scale

OHS = Oxford Hip Score

OKS = Oxford Knee Score

BMI = Body Mass Index

ASA = American Society of Anesthesiology score

NRS = Numerical Rating Scale

**Table 2:** Association between socioeconomic status and 12-month follow-up health status

	EQ-5D-3L LSS		EQ VAS		OHS/OKS		NRS Pain in rest		NRS Pain during activity	
Variables	Beta [95% CI]	*p*-value	Beta [95% CI]	*p*-value	Beta [95% CI]	*p*-value	Beta [95% CI]	*p*-value	Beta [95% CI]	*p*-value
**Total Hip Arthroplasty**										
Intercept	87.4 [86.3, 88.4]		79.9 [78.7, 81.1]		89.7 [88.7, 90.8]		89.5 [88.3, 90.7]		83.3 [81.8, 84.8]	
SES										
Q1 [least depr.]	ref		ref		ref		ref		ref	
Q2	0.2 [-0.3, 0.6]	0.512	0.4 [-0.2, 0.9]	0.186	0.3 [-0.2, 0.7]	0.283	0.0 [-0.6, 0.6]	0.997	-0.2 [-0.8, 0.5]	0.655
Q3	-0.5 [-0.9, 0.0]	0.047	-0.1 [-0.7, 0.4]	0.592	0.1 [-0.4, 0.6]	0.662	-0.5 [-1.1, 0.0]	0.053	-0.8 [-1.5, -0.2]	0.013
Q4	-0.5 [-0.9, 0.0]	0.048	-0.3 [-0.8, 0.2]	0.246	0.0 [-0.5, 0.4]	0.916	-0.8 [-1.4, -0.3]	0.001	-1.1 [-1.7, -0.4]	0.001
Q5 [most depr.]	-1.1 [-1.6, -0.7]	< 0.001	-0.8 [-1.4, -0.3]	0.003	-0.7 [-1.2, -0.2]	0.004	-1.8 [-2.4, -1.3]	< 0.001	-2.0 [-2.7, -1.4]	< 0.001
R-squared	0.10		0.10		0.09		0.03		0.02	
**Total Knee Arthroplasty**										
Intercept	81.5 [79.8, 83.1]		74.9 [73.0, 76.7]		79.3 [77.5, 81.1]		83.3 [80.9, 85.7]		73.0 [70.2, 75.8]	
SES										
Q1 [least depr.]										
Q2	0.0 [-0.6, 0.6]	0.991	0.1 [-0.6, 0.8]	0.739	-0.3 [-1.0, 0.3]	0.301	-0.9 [-1.8, -0.1]	0.037	-0.9 [-1.9, 0.1]	0.075
Q3	-0.4 [-0.9, 0.2]	0.219	-0.3 [-1.0, 0.3]	0.292	-0.3 [-0.9, 0.3]	0.381	-0.8 [-1.6, 0.0]	0.060	-0.5 [-1.5, 0.5]	0.300
Q4	-0.7 [-1.2, -0.1]	0.018	-0.6 [-1.2, 0.0]	0.050	-0.7 [-1.3, -0.1]	0.026	-1.4 [-2.2, -0.7]	< 0.001	-1.6 [-2.5, -0.7]	0.001
Q5 [most depr.]	-1.3 [-1.9, -0.7]	< 0.001	-0.5 [-1.2, 0.1]	0.108	-1.8 [-2.5, -1.2]	< 0.001	-2.7 [-3.5, -1.9]	< 0.001	-2.8 [-3.8, -1.9]	< 0.001
R-squared	0.12		0.11		0.12		0.05		0.03	

The table depicts data from the EQ-5D-3L cohort. Multivariable linear regression models were used. The LSS, OHS, OKS, and NRS outcomes were transformed to 0-100 where 100 is the best score possible. All models were adjusted for sex, age, BMI, ASA score, Charnley score, and type of hospital, and the preoperative score of the respective PROM

LSS = level sum score

VAS = Visual Analogue Scale

BMI = Body Mass Index

ASA = American Society of Anesthesiology score

OHS = Oxford Hip Score

OKS = Oxford Knee Score

NRS = Numerical Rating Scale

#### Hypothesis 2

*The negative impact of SES will be less pronounced in single-item compared to multi-item scales*.

Preoperatively, the difference between the most compared to least deprived group on the single-item EQ VAS (THA: -1.0 [95% CI -1.6, -0.4], *p* = 0.001; TKA: -0.8 [95% CI -1.5, -0.1], *p* = 0.024) was about half the size of the multi-item EQ-5D-3L LSS, and even smaller compared to the multi-item OHS/OKS. For most of these comparisons, CIs did not overlap. The differences on the single-item NRS pain scores (NRS pain in rest; THA: -3.4 [95% CI -4.2, -2.7], *p* < 0.001; TKA: -4.5 [95% CI -5.4, -3.5], *p* < 0.001) were similar to the EQ-5D-3L and OHS/OKS scores. At 12-month follow-up these patterns persisted. Moreover, at 12-month follow-up, the most vs. least deprived group of TKA patients did not report different satisfaction levels (-0.0 [95% CI -0.9, 0.8], *p* = 0.941) (Table 3). The results indicate that this hypothesis was *not met*.

**Table 3:** Association between socioeconomic status and 12-month follow-up NRS satisfaction in TKA patients

	NRS Satisfaction*	*P*-value
Variable	Beta [95% CI]	
Intercept	76.5 [74.1, 79.0]	
SES		
Q1 (least depr.)		
Q2	0.2 [-0.7, 1.1]	0.662
Q3	0.7 [-0.1, 1.6]	0.102
Q4	0.3 [-0.6, 1.1]	0.557
Q5 (most depr.)	0.0 [-0.9, 0.8]	0.941
R-squared	0.01	

The table depicts data from the EQ-5D-3L cohort. Multivariable linear regression models were used, with adjustment for sex, age, BMI, ASA score, Charnley score, and type of hospital. The NRS for satisfaction was transformed to 0-100 where 100 is the best score possible

NRS = Numerical Rating Scale

#### Hypothesis 3

*The negative impact of SES will be most pronounced when measured with the EQ-5D and OHS/OKS compared to the EQ VAS and NRS satisfaction.*

The results described under hypothesis 2 indicate that this hypothesis was *met*.

### Impact of SES on 12-month follow-up proms, stratified by preoperative health

**Fig. 2:**
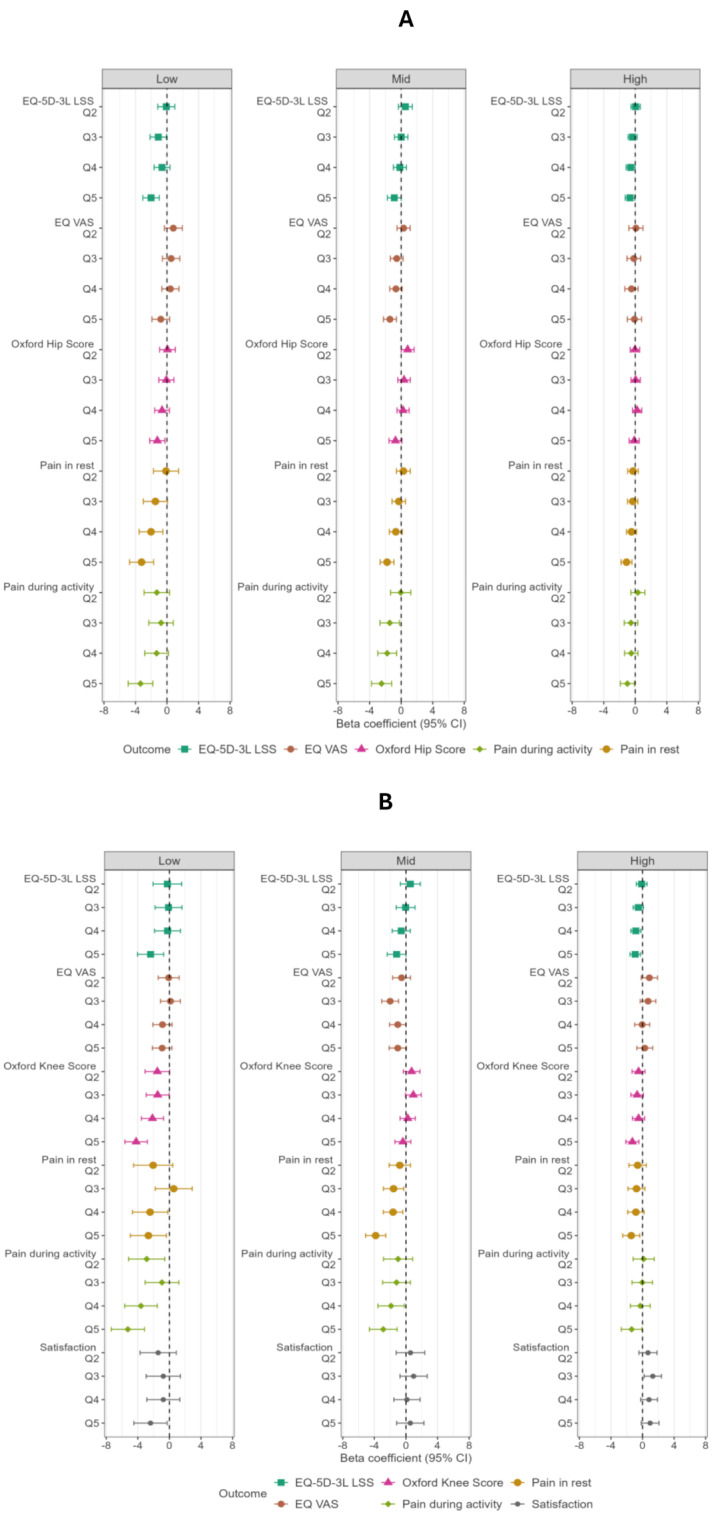
**A**: Impact of SES on 12-month follow-up outcome, stratified by preoperative outcome (THA). LSS = level sum score. VAS = Visual Analogue Scale. **B**: Impact of SES on 12-month follow-up outcome, stratified by preoperative outcome (TKA). LSS = level sum score. VAS = Visual Analogue Scale

A larger negative health difference on all PROMs between the most and least deprived groups was observed for a low preoperative score, as compared to a high preoperative score (Fig. 2A and B). This pattern seemed less pronounced for EQ VAS, particularly in TKA patients. Points-estimates of differences in NRS satisfaction in TKA patients, after stratifying according to preoperative EQ-5D-3L LSS, behaved similar to EQ VAS. Overall, these findings illustrate that the patterns described under the hypotheses seem more evident in a low vs. high preoperative score.

### Domain contribution

For the EQ-5D-3L, at each measurement point and in both THA and TKA patients, the ‘usual activities’ domain played a larger role in the SES-related PROM score differences (explained 25–46%) (Table 4). ‘Mobility’ had a limited role preoperatively (5%), which increased at 12-month follow-up (21–24%). ‘Anxiety/depression’ had a limited role in general (9–17%). Diverging patterns were seen for the other domains. For the OHS/OKS, function and pain had similar contributions to the observed differences preoperatively. At the 12-month follow-up, function (61%) was slightly more important for THA patients, while pain (68%) was more important for TKA patients.

**Table 4:** Percentage of inequality explained by each EQ-5D-3L and OHS/OKS dimension

	THA		TKA	
	Preoperative	12-month follow-up	Preoperative	12-month follow-up
**EQ-5D-L**				
Mobility	5	21	5	24
Self-care	26	36	19	13
Usual activities	37	25	31	46
Pain/discomfort	18	7	28	12
Anxiety/depression	13	13	17	9
**OHS/OKS**				
Function	46	61	38	24
Pain	54	35	62	68

The table depicts data from the EQ-5D-3L cohort. The multivariable linear regression models were used to calculate the percentage of the overall coefficient explained by each domain, as described in the methods section

OHS = Oxford Hip Score

OKS = Oxford Knee Score

### Secondary (EQ-5D-5L) cohorts

Findings regarding demographics (Supplemental File 1, Table 8, and 9), preoperative outcomes (Supplemental File 1, Table 10, and 11) and outcomes at 12-month follow-up (Supplemental File 1, Table 12, and 13) were similar between the EQ-5D-5L and EQ-5D-3L cohorts. Two exceptions were noted. First, preoperatively, inequalities between most and least deprived for the EQ-5D-5L LSS in TKA patients were smaller and insignificant (-0.78 [95% CI -1.76, 0.20]). Second, at 12-month follow-up, inequalities were smaller across all PROMs compared to the EQ-5D-3L cohort, except for OHS in THA. In the EQ-5D-5L cohort, domain contributions were roughly similar compared to the EQ-5D-3L cohort, although the earlier described pattern observed on OHS/OKS was less evident (Supplemental File 1, Table 14).

## Discussion

### Main findings

This study demonstrated the presence of SES-related health differences in Dutch THA and TKA patients, using a set of acknowledged condition-specific and generic PROMs. Higher deprivation was invariably associated with worse health, with the largest differences seen preoperatively and narrowing at 12 month follow-up. Contrary to our first hypothesis, the SES-related health differences expressed with the condition-specific OHS/OKS were similar to those in the generic EQ-5D. The second hypothesis was also rejected, as the single-item NRS pain instrument showed similar differences compared to the EQ-5D and OHS/OKS. This is most likely due to pain being the hallmark of the osteoarthritic condition, and relatively high responsiveness compensates for the limited domain coverage of the NRS for pain [21]. The single-item EQ VAS showed smaller to no SES-related health differences. We found evidence supporting the third hypothesis that the presence of reporting heterogeneity of PROMs may influence the observed inequality size. For example, NRS satisfaction did not show differences by SES groups in TKA patients at 12-month follow-up, despite health differences being present measured with PROMs assumed to exhibit less reporting heterogeneity. We are aware this evidence is not conclusive and dedicated research is needed to separate a true health difference (i.e., a similar clinical outcome in the deprived) from reporting heterogeneity (i.e., overrating by deprived patients).

Overall, the differences were relatively small, bringing into question the clinical relevance. For the OHS/OKS, a Minimal Important Difference (MID) has been established for between-group differences which may aid in determining clinical relevance [46]. Preoperative differences between the most and least deprived groups were about 25% for THA and 40% for TKA of the estimated value, with even smaller differences postoperatively. For the other PROMs, a recent study provided estimates in TKA patients that were mostly not reached before or after surgery, except for the preoperative NRS pain at rest [47]. Although the differences were mostly below MID thresholds, they may still be considered clinically and societally relevant due to ethical considerations and the large patient population involved.

### Comparison with the literature

The OHS/OKS showed a similar magnitude of outcome differences by SES compared to the generic EQ-5D, both pre- and postoperatively. This could be an indirect effect of deprivation not affecting the anxiety/depression domain of the EQ-5D. It contradicts a study conducted in the United States (US) reporting poorer mental health (PROMIS General Health Short Form) among deprived TKA patients [48]. While the anxiety/depression domain is considered sensitive [49], we presume this to be a true effect. Among the Dutch patient population, mental health problems are perhaps less likely to vary according to SES compared to the US, given the SES-indicator is categorized into quintiles. We assume the increased mental health issues in the US could be the result of steeper economic inequalities the Netherlands. Overall, we think it is reasonable to assume that the EQ-5D and OHS/OKS are measuring a similar deprivation effect, due to the considerable overlap of the domains/items that are relevant for TKA/THA patients.

### Preoperative and postoperative SES pathways

To understand the sources of SES-related inequalities, we should separate preoperative from postoperative outcome differences by SES. The largest differences by SES were observed preoperatively. The greater preoperative pain and function among the deprived suggests more severe osteoarthritis when surgery is prescribed and that different thresholds for prescribing surgery are applied. This may indicate the presence of (presumably unintentional) selection, either by the patient or the provider. A US study found that marginalized groups expressed less preference for undergoing surgery [50]. For example, Black patients perceived THA or TKA half as likely to be beneficial compared to White patients. Cost-sharing and deductibles are common features of the Dutch insurance system, which may contribute to self-selection. For example, these financial factors associate with reduced use of mental healthcare resources, potentially influencing who seeks treatment [51]. Deprived patients may also be selected at a later stage for surgery. The general practitioner at the referral stage or the surgeon during the selection procedure may show more reluctance to advise the surgery given the increased presence of comorbidity (e.g., higher BMI) [52]. Finally, communication barriers may lead to an underestimation of the pain and dysfunction levels in deprived patients at all stages [53]. In particular, more affluent patients may be able to make a stronger case for surgery. While the fact is clear-cut, the attribution of SES-related inequalities to specific underlying pathways is in its infancy, hampering reduction.

Postoperative SES-related health differences were generally smaller compared to the preoperative differences. This may suggest that the arthroplasty procedure helps mitigating healthcare inequalities. Additionally, the widespread use of standardized postoperative recovery pathways, such as Enhanced Surgical Recovery Programs, may also contribute to the fact that less health inequalities are observed postoperatively [54]. However, certain factors may continue to drive SES-related health differences. Individuals from lower SES backgrounds may face additional challenges, such as greater pressure to return to work or household duties, as well as health literacy or language barriers that hinder adherence to recovery instructions. In particular in the latter case a physiotherapist-led recovery program may be beneficial following THA/TKA. However, in the Netherlands, the first 20 physiotherapy sessions following TKA/THA are not covered by compulsory health insurance, potentially creating financial barriers for disadvantaged patients. Besides the stated explanations, it is important to acknowledge that all PROMs had some level of ceiling postoperatively, which may limit their ability to capture SES-related health differences.

### Potential reporting heterogeneity

Separating ‘true’ health differences from reporting heterogeneity, a potential source of measurement error, is challenging. We assumed that differences in EQ-5D and OHS/OKS by SES, given their wide use in THA and TKA research, best represent ‘true’ health disadvantages. The SES-related health difference was reduced/absent when measured with EQ VAS and NRS satisfaction, compared to the EQ-5D and OHS/OKS. The first explanation states that deprived patients ‘simply’ rate their overall health state to be better or are more satisfied with the result than less deprived patients, which would explain similar scores using the EQ VAS and NRS satisfaction. This explanation implies that overall health or satisfaction has the same meaning across deprivation groups. If this is the case, these measures warrant careful interpretation in the context of SES-related inequalities. Given the broad scope of these questionnaires and the lack of information on which factors influence these measurements [55], we cannot conclude that similar scores across SES categories indicate better THA/TKA care. In other words, without contextual information, these measures provide limited insight into important domains pertinent to the quality of THA/TKA, such as aspects of pain, physical and mental functioning. Although the EQ-5D and OHS/OKS lack important domains such as social functioning, they provide an explicit assessment of the abovementioned domains [56].

Another explanation for differences in the magnitude of SES-related differences is that both EQ VAS and NRS satisfaction show reporting heterogeneity [57]. In other words, the EQ VAS and NRS satisfaction measures have subtle differences in meaning and severity grading across deprivation groups. This phenomenon may turn into what has been described as the ‘disability paradox’, and entails a discrepancy between the perceived quality of life by external observers and the reported quality of life by individuals with disabilities [58]. As described earlier, phrasing may matter, and the EQ VAS and satisfaction could invite patients to respond using their own reference of the quality of care; these measures may also cover more than health care and health status alone. This internal judgment scale may be affected by prior expectations and experiences. As the EQ-5D and OHS/OKS show relatively similar inequality effects, we expect less bias in these measures. ‘Response shift’, which in essence is a change of response style over time, may also play a role. This phenomenon is described as occurring when patients undergo a process of adaptation over time, leading to a change in their internal standards, values, or conceptualization of their quality of life [59]. It is a desirable human capacity per se, enabling to live up with changed conditions, but it interferes with objective measurement. In our study, the arthroplasty procedure could be an evoking factor, and VAS/satisfaction measures could be more sensitive to this adaptation process. The best way to identify these reporting heterogeneity phenomena would be to conduct a study using vignettes (external anchors) [24].

### Strengths and limitations

We included a diverse set of PROMs, and the quality of the registry data was excellent. Moreover, the large national sample and the inclusion of both THA and TKA patients enhanced the generalizability of our findings. A key limitation is the limited information on factors that could explain SES-related health differences. We relied on postal codes to link an area-based SES indicator. Despite its proven reputation as an explanatory factor, as in our study, more specific variables such as individual-level education are also needed to delineate pathways to reduce SES-related health differences. A second limitation is our reliance on an indirect method to suggest the presence of reporting heterogeneity. Finally, while the primary and secondary cohorts yielded largely consistent findings, some discrepancies were observed. The starting year of the secondary cohort coincided with the onset of the COVID-19 pandemic, which significantly affected orthopedic care. The PROMs collected during the pandemic differed from those collected in the pre-pandemic periods, potentially accounting for the subtle differences between cohorts [60].

## Conclusions

This study demonstrated that higher deprivation in general was associated with worse health among Dutch THA and TKA patients, with the greatest differences observed preoperatively and narrowing at 12-month follow-up. The clinical relevance of these differences remains uncertain. The most significant SES-related inequalities were observed for functioning and pain (OHS/OKS, NRS pain, EQ-5D), providing potential opportunities for improvement. Overall, these findings did not translate into deprived patients rating their overall general health (EQ VAS) worse or expressing dissatisfaction (NRS satisfaction). Caution should be exercised when interpreting these latter two measures, as they may lead to overly simplistic interpretations of differences in health based on SES. Future research should focus on further identifying the drivers of inequalities and assessing whether PROMs, in particular EQ VAS and satisfaction, reflect ‘true’ health differences or are disproportionally affected by reporting heterogeneity.

## Electronic supplementary material

Below is the link to the electronic supplementary material.

Supplementary Material 1

## Data Availability

Per the agreement with the Dutch Arthroplasty Registry (LROI), the authors cannot share any data used in this study. Codes used to conduct the analyses, however, are obtainable from the corresponding author.

## Abbreviations

ASA: American Society of Anesthesiologists
BMI: Body mass index
CI: Confidence interval
LR: Linear regression
LROI: Dutch Arthroplasty Registry
LSS: Level-sum-score
NRS: Numerical Rating Scale
OHS: Oxford Hip Score
OKS: Oxford Knee Score
PROM: Patient-Reported Outcome Measure
SES: Socioeconomic status
THA: Total hip arthroplasty
TKA: Total knee arthroplasty
VAS: Visual Analogue Scale
