# Supplementary material for: Socioeconomic inequalities in patient-reported outcome measures among total hip and knee arthroplasty patients: a comprehensive analysis of instruments and domains
Source: Int J Equity Health. 2025 May 23;24:147. doi: 10.1186/s12939-025-02520-4 (PMC12102890; doi:10.1186/s12939-025-02520-4)
Supplement: Supplementary file 1 — Supplementary Material 1 [file 12939_2025_2520_MOESM1_ESM.docx]

**Supplemental File 1, Tables 1–14**

- Abbreviations: PROM = patient-reported outcome measure; THA = total hip arthroplasty; TKA = total knee arthroplasty; SES = socioeconomic status; BMI = Body Mass Index; ASA = American Society of Anesthesiology score; LSS = level-sum-score; VAS = Visual Analogue Scale; OHS = Oxford Hip Score; OKS = Oxford Knee Score; NRS = Numerical Rating Scale; IQR = Interquartile Range
- Exposure: SES, which was categorized into quintiles.
- Outcome: all PROM outcomes were transformed into a 0-100 scale where 100 represents the best attainable outcome.
- Table 1 presents cut-off values of preoperative PROM scores (tertiles).
- Table 2–3 presents descriptive statistics of demographics and outcomes of the primary EQ-5D-3L cohorts.
- Table 4–7 presents the full adjusted linear regression models studying the association between SES and respective PROM outcomes for the primary EQ-5D-3L cohorts. The regression models were adjusted for sex, age, BMI, ASA score, Charnley score, and type of hospital.
- Table 8–9 presents descriptive statistics of demographics and outcomes of the secondary EQ-5D-5L cohorts.
- Table 10–13 presents the full adjusted linear regression models studying the association between SES and respective PROM outcomes for the secondary EQ-5D-5L cohorts. The regression models were adjusted for sex, age, BMI, ASA score, Charnley score, and type of hospital.
- Table 14 presents the percentage of inequality explained by each EQ-5D-5L and OHS/OKS dimension, using data from the secondary EQ-5D-5L cohort.

**Table 1: cut-off points of tertiles of preoperative scores**

|  | **EQ-5D-3L cohort** | | | | **EQ-5D-5L cohort** | | | |
| --- | --- | --- | --- | --- | --- | --- | --- | --- |
|  | **THA** | | **TKA** | | **THA** | | **TKA** | |
| **Pre-op score LSS** | **Median (range)** | **N** | **Median (range)** | **N** | **Median (range)** | **N** | **Median (range)** | **N** |
| lowest | 50 (0, 50) | 13392 | 40 (0, 45) | 6269 | 50 (0, 50) | 3639 | 45 (0, 50) | 2216 |
| middle | 60 (60, 60) | 13100 | 55 (50, 60) | 8042 | 60 (60, 60) | 5067 | 60 (55, 65) | 3410 |
| highest | 70 (70, 100) | 19290 | 70 (65, 100) | 18423 | 70 (70, 100) | 5682 | 75 (70, 100) | 3565 |
| **Pre-op score OHS/OKS** | |  |  |  |  |  |  |  |
| lowest | 29 (0, 40) | 13893 | 33 (0, 40) | 8863 | 29 (0, 38) | 4523 | 31 (0, 40) | 2831 |
| middle | 48 (42, 54) | 12459 | 48 (42, 54) | 10478 | 48 (40, 54) | 4614 | 48 (42, 54) | 3080 |
| highest | 65 (56, 100) | 15948 | 65 (56, 100) | 10456 | 65 (56, 100) | 4872 | 65 (56, 100) | 2970 |
| **Pre-op score EQ VAS** | |  |  |  |  |  |  |  |
| lowest | 49 (0, 59) | 13392 | 50 (0, 63) | 10622 | 45 (0, 59) | 4655 | 50 (0, 60) | 2957 |
| middle | 70 (60, 77) | 16781 | 70 (64, 79) | 9951 | 70 (60, 75) | 4851 | 70 (61, 79) | 2919 |
| highest | 85 (78, 100) | 15304 | 86 (80, 100) | 11852 | 84 (76, 100) | 4793 | 85 (80, 100) | 3245 |
| **Pre-op score NRS pain in rest** | |  |  |  |  |  |  |  |
| lowest | 20 (0, 20) | 9108 | 20 (0, 20) | 6259 | 20 (0, 20) | 2983 | 20 (0, 20) | 1805 |
| middle | 40 (30, 50) | 19284 | 40 (30, 50) | 13116 | 40 (30, 50) | 6263 | 40 (30, 50) | 4002 |
| highest | 70 (60, 100) | 16816 | 70 (60, 100) | 11355 | 70 (60, 100) | 5116 | 80 (60, 100) | 3334 |
| **Pre-op score NRS pain during activity** | |  |  |  |  |  |  |  |
| lowest | 10 (0, 10) | 11392 | 10 (0, 10) | 8305 | 10 (0, 10) | 3882 | 10 (0, 10) | 2471 |
| middle | 20 (20, 20) | 13465 | 20 (20, 20) | 9288 | 20 (20, 20) | 4447 | 20 (20, 20) | 2887 |
| highest | 40 (30, 100) | 20391 | 40 (30, 100) | 13125 | 40 (30, 100) | 6016 | 40 (30, 100) | 3769 |

**Table 2: Demographics and outcomes of THA patients (EQ-5D-3L cohort)**

|  | **Q1 (least depr.)** | **Q2** | **Q3** | **Q4** | **Q5 (most depr.)** | **p-value** |
| --- | --- | --- | --- | --- | --- | --- |
| Total | 7913 | 8508 | 9804 | 10796 | 8861 |  |
| SES Z-score, median [range] | 1.3 [0.9, 2.8] | 0.6 [0.4, 0.9] | 0.2 [-0.1, 0.4] | -0.4 [-0.8, -0.1] | -1.4 [-6.3, -0.8] |  |
| Demographics |  |  |  |  |  |  |
| Age, median [IQR] | 69.0 [63.0, 75.0] | 69.0 [63.0, 75.0] | 70.0 [63.0, 75.0] | 70.0 [63.0, 75.0] | 70.0 [64.0, 76.0] | <0.001 |
| <50 | 271 (3) | 258 (3) | 262 (3) | 283 (3) | 226 (3) | <0.001 |
| 50-69 | 3739 (47) | 4108 (48) | 4631 (47) | 5088 (47) | 3930 (44) |  |
| >70 | 3903 (49) | 4142 (49) | 4911 (50) | 5425 (50) | 4705 (53) |  |
| BMI, median [IQR] | 26.1 [23.9, 29.0] | 26.6 [24.1, 29.7] | 26.9 [24.3, 29.8] | 27.0 [24.4, 30.0] | 27.3 [24.7, 30.4] | <0.001 |
| <25 | 3400 (43) | 3278 (39) | 3570 (36) | 3793 (35) | 2876 (32) | <0.001 |
| 25-30 | 3162 (40) | 3496 (41) | 4173 (43) | 4600 (43) | 3817 (43) |  |
| >30 | 1351 (17) | 1734 (20) | 2061 (21) | 2403 (22) | 2168 (24) |  |
| Male | 5020 (63) | 5329 (63) | 6155 (63) | 6964 (65) | 5817 (66) | 0.003 |
| ASA |  |  |  |  |  | <0.001 |
| I | 1749 (22) | 1744 (20) | 1960 (20) | 1874 (17) | 1303 (15) |  |
| II | 4997 (63) | 5489 (65) | 6260 (64) | 7078 (66) | 5776 (65) |  |
| III-IV | 1167 (15) | 1275 (15) | 1584 (16) | 1844 (17) | 1782 (20) |  |
| Charnley |  |  |  |  |  | <0.001 |
| A | 3619 (46) | 3714 (44) | 4371 (45) | 4584 (42) | 3907 (44) |  |
| B1 | 2497 (32) | 2695 (32) | 2987 (30) | 3530 (33) | 2693 (30) |  |
| B2 | 1634 (21) | 1871 (22) | 2118 (22) | 2324 (22) | 1942 (22) |  |
| C | 163 (2) | 228 (3) | 328 (3) | 358 (3) | 319 (4) |  |
| Smoking (yes) | 600 (8) | 766 (9) | 928 (9) | 986 (9) | 896 (10) | <0.001 |
| Previous surgery of the joint (yes) | 135 (2) | 130 (2) | 134 (1) | 183 (2) | 162 (2) | 0.589 |
| Year of surgery |  |  |  |  |  | <0.001 |
| 2014 | 426 (5) | 372 (4) | 433 (4) | 426 (4) | 452 (5) |  |
| 2015 | 1034 (13) | 1026 (12) | 1142 (12) | 1119 (10) | 941 (11) |  |
| 2016 | 1338 (17) | 1492 (18) | 1710 (17) | 1776 (16) | 1433 (16) |  |
| 2017 | 1546 (20) | 1685 (20) | 1986 (20) | 2164 (20) | 1811 (20) |  |
| 2018 | 1748 (22) | 1884 (22) | 2202 (22) | 2420 (22) | 1955 (22) |  |
| 2019 | 1468 (19) | 1686 (20) | 1974 (20) | 2300 (21) | 1736 (20) |  |
| 2020 | 243 (3) | 233 (3) | 222 (2) | 323 (3) | 343 (4) |  |
| 2021 | 77 (1) | 82 (1) | 102 (1) | 193 (2) | 145 (2) |  |
| 2022 | 33 (0) | 48 (1) | 33 (0) | 75 (1) | 45 (1) |  |
| Type of hospital |  |  |  |  |  | <0.001 |
| General Hospital | 7013 (89) | 7559 (89) | 8822 (90) | 9836 (91) | 8099 (91) |  |
| Private Hospital | 819 (10) | 817 (10) | 852 (9) | 817 (8) | 493 (6) |  |
| University Medical Center | 81 (1) | 132 (2) | 130 (1) | 143 (1) | 269 (3) |  |
| Fixation |  |  |  |  |  | <0.001 |
| Cemented | 1441 (18) | 1605 (19) | 1927 (20) | 2338 (22) | 2470 (28) |  |
| Cementless | 5918 (75) | 6152 (72) | 6882 (70) | 7549 (70) | 5510 (62) |  |
| Hybrid | 554 (7) | 751 (9) | 995 (10) | 909 (8) | 881 (10) |  |
| Approach |  |  |  |  |  | <0.001 |
| Anterior | 3458 (44) | 3393 (40) | 3462 (35) | 3546 (33) | 2830 (32) |  |
| Anterolateral | 235 (3) | 341 (4) | 414 (4) | 445 (4) | 418 (5) |  |
| Other | 78 (1) | 64 (1) | 114 (1) | 193 (2) | 105 (1) |  |
| Posterolateral | 3608 (46) | 4121 (48) | 5262 (54) | 5997 (56) | 4945 (56) |  |
| Straight lateral | 534 (7) | 589 (7) | 552 (6) | 615 (6) | 563 (6) |  |
| Contralateral procedure (yes) | 760 (10) | 879 (10) | 1024 (10) | 1152 (11) | 900 (10) | 0.242 |
| Outcomes |  |  |  |  |  |  |
| Preoperative outcomes, median [IQR] |  |  |  |  |  |  |
| EQ-5D-3L LSS | 60.0 [50.0, 70.0] | 60.0 [50.0, 70.0] | 60.0 [50.0, 70.0] | 60.0 [50.0, 70.0] | 60.0 [50.0, 70.0] |  |
| OHS | 52.1 [37.5, 62.5] | 50.0 [37.5, 62.5] | 50.0 [35.4, 60.4] | 47.9 [35.4, 60.4] | 47.9 [33.3, 60.4] |  |
| EQ VAS | 70.0 [56.0, 80.0] | 70.0 [54.0, 80.0] | 70.0 [53.0, 80.0] | 70.0 [53.0, 80.0] | 70.0 [51.0, 80.0] |  |
| NRS Pain in rest | 50.0 [30.0, 70.0] | 50.0 [30.0, 70.0] | 50.0 [30.0, 70.0] | 40.0 [30.0, 70.0] | 40.0 [30.0, 60.0] |  |
| NRS Pain during activity | 20.0 [20.0, 40.0] | 20.0 [20.0, 40.0] | 20.0 [10.0, 40.0] | 20.0 [10.0, 40.0] | 20.0 [10.0, 40.0] |  |
| 12-month follow-up outcomes, median [IQR] |  |  |  |  |  |  |
| EQ-5D-3L LSS | 100.0 [80.0, 100.0] | 90.0 [80.0, 100.0] | 90.0 [80.0, 100.0] | 90.0 [80.0, 100.0] | 90.0 [70.0, 100.0] |  |
| OHS | 93.8 [83.3, 97.9] | 93.8 [83.3, 97.9] | 93.8 [83.3, 97.9] | 93.8 [83.3, 97.9] | 91.7 [81.2, 97.9] |  |
| EQ VAS | 80.0 [70.0, 90.0] | 80.0 [70.0, 90.0] | 80.0 [70.0, 90.0] | 80.0 [70.0, 90.0] | 80.0 [70.0, 90.0] |  |
| NRS Pain in rest | 100.0 [90.0, 100.0] | 100.0 [90.0, 100.0] | 100.0 [90.0, 100.0] | 100.0 [90.0, 100.0] | 100.0 [90.0, 100.0] | |
| NRS Pain during activity | 100.0 [80.0, 100.0] | 100.0 [80.0, 100.0] | 100.0 [80.0, 100.0] | 100.0 [80.0, 100.0] | 90.0 [80.0, 100.0] |  |
| Preoperative outcomes, n (%) ceiling |  |  |  |  |  |  |
| EQ-5D-3L LSS | 68 (0.9) | 79 (0.9) | 66 (0.7) | 54 (0.5) | 68 (0.8) |  |
| OHS | 9 (0.1) | 3 (0.0) | 2 (0.0) | 3 (0.0) | 3 (0.0) |  |
| EQ VAS | 151 (1.9) | 124 (1.5) | 173 (1.8) | 185 (1.7) | 167 (1.9) |  |
| NRS Pain in rest | 432 (5.5) | 414 (5.0) | 468 (4.9) | 500 (4.7) | 446 (5.1) |  |
| NRS Pain during activity | 71 (0.9) | 76 (0.9) | 84 (0.9) | 90 (0.8) | 95 (1.1) |  |
| 12-month follow-up outcomes, n (%) ceiling |  |  |  |  |  |  |
| EQ-5D-3L LSS | 3978 (50.3) | 4227 (49.7) | 4705 (48.0) | 5023 (46.5) | 3924 (44.3) |  |
| OHS | 1728 (24.7) | 1824 (23.6) | 2104 (23.6) | 2150 (21.7) | 1545 (20.0) |  |
| EQ VAS | 480 (6.1) | 570 (6.7) | 718 (7.4) | 715 (6.7) | 618 (7.0) |  |
| NRS Pain in rest | 5426 (70.4) | 5743 (68.9) | 6591 (68.6) | 7192 (67.4) | 5679 (65.1) |  |
| NRS Pain during activity | 4190 (54.4) | 4440 (53.2) | 5053 (52.5) | 5460 (51.2) | 4346 (49.7) |  |

**Table 3: Demographics and outcomes of TKA patients (EQ-5D-3L cohort)**

|  | **Q1 (least depr.)** | **Q2** | **Q3** | **Q4** | **Q5 (most depr.)** | **p-value** |
| --- | --- | --- | --- | --- | --- | --- |
| Total | 4860 | 5589 | 6990 | 8335 | 6960 |  |
| SES Z-score, median [range] | 1.3 [0.9, 2.7] | 0.6 [0.4, 0.9] | 0.2 [-0.1, 0.4] | -0.4 [-0.8, -0.1] | -1.4 [-6.2, -0.8] |  |
| Demographics |  |  |  |  |  |  |
| Age, median [IQR] | 69.0 [63.0, 75.0] | 69.0 [63.0, 74.0] | 69.0 [63.0, 74.0] | 69.0 [63.0, 74.0] | 69.0 [63.0, 75.0] | 0.373 |
| <50 | 49 (1) | 80 (1) | 93 (1) | 119 (1) | 93 (1) | 0.105 |
| 50-69 | 2386 (49) | 2915 (52) | 3706 (53) | 4355 (52) | 3495 (50) |  |
| >70 | 2425 (50) | 2594 (46) | 3191 (46) | 3861 (46) | 3372 (48) |  |
| BMI, median [IQR] | 28.4 [25.7, 31.7] | 28.7 [25.9, 32.0] | 28.8 [26.0, 32.2] | 29.0 [26.2, 32.7] | 29.4 [26.4, 33.2] | <0.001 |
| <25 | 1101 (23) | 1184 (21) | 1444 (21) | 1585 (19) | 1233 (18) | <0.001 |
| 25-30 | 2114 (43) | 2456 (44) | 2928 (42) | 3508 (42) | 2777 (40) |  |
| >30 | 1645 (34) | 1949 (35) | 2618 (37) | 3242 (39) | 2950 (42) |  |
| Male |  |  |  |  |  | 0.009 |
| ASA |  |  |  |  |  | <0.001 |
| I | 738 (15) | 826 (15) | 1042 (15) | 1085 (13) | 705 (10) |  |
| II | 3284 (68) | 3794 (68) | 4697 (67) | 5598 (67) | 4657 (67) |  |
| III-IV | 838 (17) | 969 (17) | 1251 (18) | 1652 (20) | 1598 (23) |  |
| Charnley |  |  |  |  |  | 0.154 |
| A | 2053 (42) | 2415 (43) | 2967 (42) | 3479 (42) | 2948 (42) |  |
| B1 | 1682 (35) | 1851 (33) | 2329 (33) | 2798 (34) | 2306 (33) |  |
| B2 | 991 (20) | 1114 (20) | 1442 (21) | 1728 (21) | 1479 (21) |  |
| C | 134 (3) | 209 (4) | 252 (4) | 330 (4) | 227 (3) |  |
| Smoking (yes) | 288 (6) | 381 (7) | 518 (7) | 646 (8) | 614 (9) | <0.001 |
| Previous surgery of the joint (yes) | 1370 (28) | 1687 (30) | 2146 (31) | 2595 (31) | 1929 (28) | 0.586 |
| Year of surgery |  |  |  |  |  | <0.001 |
| 2014 | 78 (2) | 64 (1) | 66 (1) | 54 (1) | 54 (1) |  |
| 2015 | 329 (7) | 307 (5) | 336 (5) | 417 (5) | 325 (5) |  |
| 2016 | 863 (18) | 1045 (19) | 1367 (20) | 1567 (19) | 1313 (19) |  |
| 2017 | 1254 (26) | 1357 (24) | 1761 (25) | 2077 (25) | 1768 (25) |  |
| 2018 | 1124 (23) | 1337 (24) | 1664 (24) | 2052 (25) | 1634 (23) |  |
| 2019 | 948 (20) | 1212 (22) | 1455 (21) | 1698 (20) | 1462 (21) |  |
| 2020 | 172 (4) | 155 (3) | 203 (3) | 269 (3) | 258 (4) |  |
| 2021 | 61 (1) | 89 (2) | 111 (2) | 146 (2) | 105 (2) |  |
| 2022 | 31 (1) | 23 (0) | 27 (0) | 55 (1) | 41 (1) |  |
| Type of hospital |  |  |  |  |  | <0.001 |
| General Hospital | 4202 (86) | 4820 (86) | 6146 (88) | 7506 (90) | 6316 (91) |  |
| Private Hospital | 602 (12) | 708 (13) | 784 (11) | 757 (9) | 484 (7) |  |
| University Medical Center | 56 (1) | 61 (1) | 60 (1) | 72 (1) | 160 (2) |  |
| Fixation |  |  |  |  |  | <0.001 |
| Cemented | 4338 (89) | 5292 (95) | 6644 (95) | 7806 (94) | 6473 (93) |  |
| Cementless | 346 (7) | 119 (2) | 211 (3) | 276 (3) | 234 (3) |  |
| Hybrid | 176 (4) | 178 (3) | 135 (2) | 253 (3) | 253 (4) |  |
| Approach |  |  |  |  |  | <0.001 |
| Lateral parapatellar | 35 (1) | 31 (1) | 34 (0) | 39 (0) | 34 (0) |  |
| Medial parapatellar | 4697 (97) | 5426 (97) | 6784 (97) | 8153 (98) | 6834 (98) |  |
| Other | 3 (0) | 5 (0) | 4 (0) | 9 (0) | 6 (0) |  |
| Vastus (mid/sub) | 125 (3) | 127 (2) | 168 (2) | 134 (2) | 86 (1) |  |
| Contralateral procedure (yes) | 565 (12) | 694 (12) | 892 (13) | 1101 (13) | 890 (13) | 0.062 |
| Outcomes |  |  |  |  |  |  |
| Preoperative outcomes, median [IQR] |  |  |  |  |  |  |
| EQ-5D-3L LSS | 70.0 [60.0, 70.0] | 70.0 [60.0, 70.0] | 70.0 [60.0, 70.0] | 70.0 [60.0, 70.0] | 70.0 [60.0, 70.0] |  |
| OKS | 52.1 [39.6, 62.5] | 50.0 [39.6, 60.4] | 50.0 [39.6, 60.4] | 47.9 [37.5, 58.3] | 47.9 [35.4, 58.3] |  |
| EQ VAS | 72.0 [60.0, 82.0] | 73.0 [60.0, 83.0] | 72.0 [60.0, 81.0] | 71.0 [60.0, 80.0] | 70.0 [56.0, 80.0] |  |
| NRS Pain in rest | 50.0 [30.0, 70.0] | 50.0 [30.0, 70.0] | 50.0 [30.0, 70.0] | 40.0 [30.0, 70.0] | 40.0 [30.0, 60.0] |  |
| NRS Pain during activity | 20.0 [20.0, 40.0] | 20.0 [20.0, 40.0] | 20.0 [10.0, 30.0] | 20.0 [10.0, 30.0] | 20.0 [10.0, 30.0] |  |
| 12-month follow-up outcomes, median [IQR] |  |  |  |  |  |  |
| EQ-5D-3L LSS | 90.0 [70.0, 100.0] | 90.0 [70.0, 100.0] | 90.0 [70.0, 100.0] | 90.0 [70.0, 100.0] | 90.0 [70.0, 100.0] |  |
| OKS | 87.5 [75.0, 95.8] | 87.5 [75.0, 93.8] | 87.5 [75.0, 93.8] | 85.4 [72.9, 93.8] | 83.3 [68.8, 93.8] |  |
| EQ VAS | 80.0 [70.0, 90.0] | 80.0 [70.0, 90.0] | 80.0 [70.0, 90.0] | 80.0 [70.0, 90.0] | 80.0 [67.0, 90.0] |  |
| NRS Pain in rest | 100.0 [80.0, 100.0] | 100.0 [80.0, 100.0] | 100.0 [80.0, 100.0] | 90.0 [80.0, 100.0] | 90.0 [70.0, 100.0] |  |
| NRS Pain during activity | 90.0 [70.0, 100.0] | 90.0 [60.0, 100.0] | 90.0 [60.0, 100.0] | 80.0 [60.0, 100.0] | 80.0 [60.0, 100.0] |  |
| NRS Satisfaction | 80.0 [70.0, 100.0] | 80.0 [70.0, 100.0] | 80.0 [70.0, 100.0] | 80.0 [70.0, 90.0] | 80.0 [70.0, 100.0] |  |
| Preoperative outcomes, n (%) ceiling |  |  |  |  |  |  |
| EQ-5D-3L LSS | 64 (1.3) | 75 (1.3) | 73 (1.0) | 72 (0.9) | 65 (0.9) |  |
| OKS | 2 (0.0) | 3 (0.1) | 4 (0.1) | 2 (0.0) | 0 (0.0) |  |
| EQ VAS | 92 (1.9) | 119 (2.2) | 136 (2.0) | 173 (2.1) | 148 (2.1) |  |
| NRS Pain in rest | 307 (6.8) | 294 (5.6) | 345 (5.3) | 398 (5.1) | 355 (5.3) |  |
| NRS Pain during activity | 39 (0.9) | 38 (0.7) | 49 (0.8) | 56 (0.7) | 80 (1.2) |  |
| 12-month follow-up outcomes, n (%) ceiling |  |  |  |  |  |  |
| EQ-5D-3L LSS | 2069 (42.6) | 2346 (42.0) | 2852 (40.8) | 3314 (39.8) | 2578 (37.0) |  |
| OKS | 344 (8.0) | 358 (7.1) | 440 (6.9) | 535 (7.0) | 370 (5.9) |  |
| EQ VAS | 214 (4.4) | 252 (4.6) | 326 (4.7) | 383 (4.6) | 307 (4.5) |  |
| NRS Pain in rest | 2498 (54.6) | 2784 (51.7) | 3454 (51.5) | 4029 (49.8) | 3282 (48.1) |  |
| NRS Pain during activity | 1586 (34.6) | 1802 (33.4) | 2238 (33.3) | 2617 (32.3) | 2146 (31.5) |  |
| NRS Satisfaction | 1075 (26.2) | 1284 (25.6) | 1575 (25.2) | 1877 (24.9) | 1540 (25.0) |  |

**Table 4: Association between socioeconomic status and preoperative health status of THA patients (EQ-5D-3L cohort)**

|  | **LSS** | **VAS** | **OHS** | **NRS Pain in rest** | **NRS Pain during activity** |
| --- | --- | --- | --- | --- | --- |
| **Variables** | **Coefficient (95% CI)** | **Coefficient (95% CI)** | **Coefficient (95% CI)** | **Coefficient (95% CI)** | **Coefficient (95% CI)** |
| Intercept | 61.48 (60.56, 62.40) | 67.43 (66.15, 68.72) | 50.52 (49.33, 51.71) | 45.07 (43.38, 46.75) | 25.54 (24.20, 26.89) |
| SES |  |  |  |  |  |
| Q1, least deprived |  |  |  |  |  |
| Q2 | -0.53 (-0.96, -0.11) | -0.84 (-1.42, -0.25) | -0.65 (-1.20, -0.11) | -1.04 (-1.81, -0.26) | -0.73 (-1.34, -0.11) |
| Q3 | -1.07 (-1.48, -0.67) | -0.77 (-1.34, -0.20) | -1.35 (-1.88, -0.83) | -1.72 (-2.46, -0.97) | -1.16 (-1.76, -0.56) |
| Q4 | -1.40 (-1.80, -1.00) | -0.56 (-1.12, 0.00) | -1.84 (-2.35, -1.33) | -2.35 (-3.08, -1.62) | -2.13 (-2.71, -1.54) |
| Q5, most deprived | -1.62 (-2.04, -1.20) | -0.97 (-1.56, -0.39) | -2.42 (-2.96, -1.88) | -3.42 (-4.19, -2.65) | -1.54 (-2.16, -0.93) |
| Male (vs. female) | 2.93 (2.67, 3.20) | 3.71 (3.34, 4.08) | 5.42 (5.08, 5.76) | 5.30 (4.82, 5.79) | 4.06 (3.68, 4.45) |
| Age |  |  |  |  |  |
| <50 |  |  |  |  |  |
| 50-69 | 4.26 (3.49, 5.04) | 4.62 (3.54, 5.71) | 3.74 (2.73, 4.74) | 5.14 (3.71, 6.56) | 3.93 (2.79, 5.06) |
| >70 | 4.33 (3.54, 5.11) | 6.72 (5.62, 7.81) | 3.68 (2.67, 4.70) | 8.78 (7.34, 10.22) | 5.55 (4.40, 6.70) |
| BMI |  |  |  |  |  |
| <25 |  |  |  |  |  |
| 25-30 | -1.20 (-1.48, -0.91) | -0.96 (-1.37, -0.56) | -2.62 (-2.99, -2.25) | -2.37 (-2.90, -1.85) | -1.93 (-2.35, -1.51) |
| >30 | -3.32 (-3.68, -2.96) | -3.36 (-3.86, -2.86) | -6.42 (-6.87, -5.96) | -4.32 (-4.98, -3.67) | -3.97 (-4.49, -3.45) |
| ASA |  |  |  |  |  |
| I |  |  |  |  |  |
| II | -2.29 (-2.64, -1.95) | -3.66 (-4.15, -3.18) | -2.87 (-3.31, -2.43) | -2.19 (-2.82, -1.56) | -2.46 (-2.96, -1.95) |
| III-IV | -6.89 (-7.35, -6.43) | -9.26 (-9.90, -8.62) | -8.22 (-8.81, -7.63) | -4.50 (-5.34, -3.66) | -5.70 (-6.37, -5.03) |
| Charnley |  |  |  |  |  |
| A |  |  |  |  |  |
| B1 | 0.00 (-0.30, 0.29) | 0.23 (-0.18, 0.64) | 0.57 (0.19, 0.95) | -0.06 (-0.60, 0.48) | 0.17 (-0.26, 0.60) |
| B2 | 1.29 (0.96, 1.62) | 0.73 (0.27, 1.20) | 1.78 (1.36, 2.21) | -0.34 (-0.95, 0.27) | 1.47 (0.99, 1.96) |
| C | -1.45 (-2.21, -0.70) | -1.17 (-2.23, -0.12) | -1.82 (-2.77, -0.88) | -3.68 (-5.05, -2.30) | -0.67 (-1.77, 0.43) |
| Hospital |  |  |  |  |  |
| Private Hospital |  |  |  |  |  |
| General Hospital | -0.98 (-1.45, -0.50) | -2.45 (-3.11, -1.79) | -0.99 (-1.58, -0.39) | 0.76 (-0.10, 1.62) | 1.74 (1.06, 2.43) |
| University Medical Center | -5.71 (-6.80, -4.63) | -5.45 (-6.97, -3.94) | -9.74 (-11.18, -8.30) | -2.03 (-4.04, -0.02) | -2.03 (-3.63, -0.42) |
| R-squared | 0.053 | 0.040 | 0.078 | 0.026 | 0.027 |

**Table 5: Association between socioeconomic status and preoperative health status of TKA patients (EQ-5D-3L cohort)**

|  | **LSS** | **VAS** | **OKS** | **NRS Pain in rest** | **NRS Pain during activity** |
| --- | --- | --- | --- | --- | --- |
| **Variables** | **Coefficient (95% CI)** | **Coefficient (95% CI)** | **Coefficient (95% CI)** | **Coefficient (95% CI)** | **Coefficient (95% CI)** |
| Intercept | 65.44 (64.07, 66.81) | 71.39 (69.44, 73.35) | 51.16 (49.00 ,53.32) | 45.72 (42.97, 48.48) | 26.00 (23.86, 28.15) |
| SES |  |  |  |  |  |
| Q1, least deprived |  |  |  |  |  |
| Q2 | -0.51 (-1.00, -0.01) | 0.58 (-0.13, 1.28) | -1.44 (-2.37 ,-0.52) | -0.81 (-1.81, 0.19) | -0.67 (-1.44, 0.11) |
| Q3 | -1.22 (-1.69, -0.74) | 0.26 (-0.41, 0.94) | -1.96 (-2.87 ,-1.06) | -2.54 (-3.50, -1.59) | -1.77 (-2.52, -1.03) |
| Q4 | -1.52 (-1.97, -1.06) | 0.15 (-0.50, 0.80) | -1.94 (-2.84 ,-1.04) | -3.37 (-4.29, -2.45) | -2.02 (-2.74, -1.31) |
| Q5, most deprived | -2.07 (-2.55, -1.60) | -0.78 (-1.46, -0.10) | -4.33 (-5.28 ,-3.39) | -4.47 (-5.42, -3.51) | -1.89 (-2.63, -1.15) |
| Male (vs. female) | 3.07 (2.78, 3.36) | 3.62 (3.20, 4.03) | 4.80 (4.20 ,5.39) | 5.01 (4.43, 5.60) | 3.68 (3.22, 4.13) |
| Age |  |  |  |  |  |
| <50 |  |  |  |  |  |
| 50-69 | 4.10 (2.87, 5.34) | 4.56 (2.81, 6.32) | 4.05 (2.06 ,6.03) | 4.19 (1.72, 6.66) | 3.17 (1.24, 5.09) |
| >70 | 5.01 (3.77, 6.25) | 6.41 (4.64, 8.17) | 4.20 (2.20 ,6.21) | 9.26 (6.78, 11.75) | 6.07 (4.13, 8.00) |
| BMI |  |  |  |  |  |
| <25 |  |  |  |  |  |
| 25-30 | -0.93 (-1.31, -0.55) | -0.56 (-1.10, -0.02) | -3.16 (-3.81 ,-2.52) | -2.08 (-2.85, -1.31) | -1.39 (-1.98, -0.79) |
| >30 | -2.70 (-3.10, -2.30) | -2.74 (-3.31, -2.16) | -8.24 (-9.05 ,-7.42) | -4.93 (-5.74, -4.12) | -3.52 (-4.15, -2.89) |
| ASA |  |  |  |  |  |
| I |  |  |  |  |  |
| II | -2.14 (-2.58, -1.70) | -4.24 (-4.87, -3.62) | -3.09 (-3.91 ,-2.27) | -0.81 (-1.70, 0.07) | -1.62 (-2.31, -0.93) |
| III-IV | -5.70 (-6.24, -5.16) | -9.78 (-10.54, -9.01) | -8.03 (-9.07 ,-6.98) | -2.74 (-3.82, -1.66) | -4.12 (-4.97, -3.28) |
| Charnley |  |  |  |  |  |
| A |  |  |  |  |  |
| B1 | -0.14 (-0.47, 0.18) | -0.40 (-0.86, 0.06) | 0.65 (-0.02 ,1.31) | 0.22 (-0.44, 0.87) | -0.61 (-1.12, -0.10) |
| B2 | 1.48 (1.11, 1.86) | 1.25 (0.71, 1.79) | 1.89 (1.14 ,2.63) | 0.76 (0.00, 1.52) | 1.63 (1.04, 2.23) |
| C | -2.20 (-2.98, -1.42) | -0.64 (-1.74, 0.47) | -1.52 (-3.20 ,0.17) | -1.43 (-3.01, 0.15) | -0.84 (-2.07, 0.39) |
| Hospital |  |  |  |  |  |
| Private Hospital |  |  |  |  |  |
| General Hospital | -1.24 (-1.73, -0.76) | -3.32 (-4.01, -2.63) | -2.09 (-2.80 ,-1.38) | 0.49 (-0.51, 1.50) | 0.41 (-0.38, 1.19) |
| University Medical Center | -3.51 (-4.85, -2.18) | -4.03 (-5.95, -2.11) | -5.02 (-9.78 ,-0.25) | -0.20 (-2.96, 2.56) | 0.22 (-1.93, 2.36) |
| R-squared | 0.053 | 0.051 | 0.089 | 0.033 | 0.028 |

**Table 6: Association between socioeconomic status and 12-month follow-up health status of THA patients (EQ-5D-3L cohort)**

|  | **LSS** | **VAS** | **OHS** | **NRS Pain in rest** | **NRS Pain during activity** |
| --- | --- | --- | --- | --- | --- |
| **Variables** | **Coefficient (95% CI)** | **Coefficient (95% CI)** | **Coefficient (95% CI)** | **Coefficient (95% CI)** | **Coefficient (95% CI)** |
| Intercept | 87.38 (86.33, 88.44) | 79.93 (78.71, 81.14) | 89.74 (88.71 ,90.77) | 89.50 (88.26, 90.74) | 83.28 (81.76, 84.80) |
| SES |  |  |  |  |  |
| Q1, least deprived |  |  |  |  |  |
| Q2 | 0.16 (-0.31, 0.63) | 0.37 (-0.18, 0.91) | 0.25 (-0.21 ,0.71) | 0.00 (-0.55, 0.55) | -0.15 (-0.83, 0.52) |
| Q3 | -0.46 (-0.92, -0.01) | -0.14 (-0.67, 0.38) | 0.10 (-0.35 ,0.55) | -0.52 (-1.06, 0.01) | -0.84 (-1.50, -0.18) |
| Q4 | -0.45 (-0.90, 0.00) | -0.31 (-0.82, 0.21) | -0.02 (-0.46 ,0.41) | -0.84 (-1.36, -0.32) | -1.05 (-1.69, -0.41) |
| Q5, most deprived | -1.14 (-1.61, -0.67) | -0.83 (-1.37, -0.29) | -0.69 (-1.15 ,-0.22) | -1.83 (-2.38, -1.29) | -2.03 (-2.71, -1.35) |
| Pre-op score in 3 categories |  |  |  |  |  |
| lowest |  |  |  |  |  |
| middle | 5.06 (4.69, 5.43) | 4.34 (3.94, 4.75) | 3.64 (3.29 ,4.00) | 2.91 (2.47, 3.36) | 2.11 (1.56, 2.66) |
| highest | 8.44 (8.10, 8.79) | 9.88 (9.46, 10.30) | 6.20 (5.86 ,6.54) | 7.03 (6.57, 7.49) | 4.68 (4.17, 5.19) |
| Male (vs. female) | 2.70 (2.40, 3.00) | 2.01 (1.67, 2.36) | 1.99 (1.70 ,2.29) | 1.13 (0.79, 1.48) | 1.95 (1.52, 2.38) |
| Age |  |  |  |  |  |
| <50 |  |  |  |  |  |
| 50-69 | 0.87 (0.01, 1.74) | -0.09 (-1.08, 0.91) | 0.65 (-0.20 ,1.51) | 2.11 (1.10, 3.12) | 3.73 (2.49, 4.98) |
| >70 | -0.42 (-1.30, 0.46) | -2.25 (-3.26, -1.24) | -0.96 (-1.83 ,-0.10) | 1.56 (0.54, 2.58) | 4.24 (2.97, 5.50) |
| BMI |  |  |  |  |  |
| <25 |  |  |  |  |  |
| 25-30 | -1.31 (-1.63, -0.99) | -1.10 (-1.47, -0.73) | -1.34 (-1.65 ,-1.02) | -0.76 (-1.14, -0.39) | -1.53 (-2.00, -1.07) |
| >30 | -3.28 (-3.68, -2.88) | -2.79 (-3.25, -2.33) | -3.41 (-3.81 ,-3.02) | -1.59 (-2.05, -1.12) | -2.86 (-3.44, -2.29) |
| ASA |  |  |  |  |  |
| I |  |  |  |  |  |
| II | -2.54 (-2.92, -2.15) | -3.35 (-3.79, -2.90) | -1.95 (-2.33 ,-1.58) | -1.78 (-2.23, -1.33) | -2.16 (-2.72, -1.60) |
| III-IV | -6.32 (-6.83, -5.80) | -8.17 (-8.77, -7.57) | -5.44 (-5.94 ,-4.93) | -3.23 (-3.83, -2.64) | -4.11 (-4.85, -3.37) |
| Charnley |  |  |  |  |  |
| A |  |  |  |  |  |
| B1 | -0.49 (-0.82, -0.16) | -0.19 (-0.57, 0.19) | -0.46 (-0.78 ,-0.13) | 0.07 (-0.31, 0.45) | 0.28 (-0.19, 0.76) |
| B2 | -1.13 (-1.50, -0.76) | -0.47 (-0.90, -0.04) | -0.58 (-0.95 ,-0.22) | 0.39 (-0.04, 0.82) | 0.87 (0.33, 1.40) |
| C | -2.63 (-3.48, -1.79) | -2.28 (-3.26, -1.30) | -1.54 (-2.35 ,-0.73) | -0.71 (-1.69, 0.27) | -0.86 (-2.07, 0.35) |
| Hospital |  |  |  |  |  |
| Private Hospital |  |  |  |  |  |
| General Hospital | -2.06 (-2.58, -1.53) | -2.03 (-2.64, -1.42) | -2.06 (-2.56 ,-1.55) | -1.68 (-2.29, -1.08) | -1.48 (-2.23, -0.72) |
| University Medical Center | -5.96 (-7.17, -4.74) | -3.76 (-5.16, -2.36) | -5.14 (-6.36 ,-3.92) | -1.29 (-2.73, 0.15) | -2.79 (-4.58, -1.01) |
| R-squared | 0.103 | 0.095 | 0.085 | 0.034 | 0.021 |

**Table 7: Association between socioeconomic status and 12-month follow-up health status of TKA patients (EQ-5D-3L cohort)**

|  | **LSS** | **VAS** | **OKS** | **NRS Pain in rest** | **NRS Pain during activity** | **Satisfaction** |
| --- | --- | --- | --- | --- | --- | --- |
| **Variables** | **Coefficient (95% CI)** | **Coefficient (95% CI)** | **Coefficient (95% CI)** | **Coefficient (95% CI)** | **Coefficient (95% CI)** | **Coefficient (95% CI)** |
| Intercept | 81.46 (79.78, 83.13) | 74.85 (72.97, 76.72) | 79.31 (77.53 ,81.09) | 83.28 (80.87, 85.69) | 73.02 (70.22, 75.82) | 76.52 (74.06, 78.97) |
| SES |  |  |  |  |  |  |
| Q1, least deprived |  |  |  |  |  |  |
| Q2 | 0.00 (-0.60, 0.59) | 0.11 (-0.56, 0.78) | -0.34 (-0.98 ,0.30) | -0.91 (-1.76, -0.05) | -0.91 (-1.91, 0.09) | 0.20 (-0.70, 1.10) |
| Q3 | -0.35 (-0.92, 0.21) | -0.34 (-0.98, 0.30) | -0.27 (-0.89 ,0.34) | -0.78 (-1.60, 0.03) | -0.50 (-1.46, 0.45) | 0.71 (-0.14, 1.57) |
| Q4 | -0.66 (-1.21, -0.12) | -0.62 (-1.24, 0.00) | -0.67 (-1.27 ,-0.08) | -1.44 (-2.23, -0.65) | -1.58 (-2.50, -0.66) | 0.25 (-0.58, 1.08) |
| Q5, most deprived | -1.30 (-1.87, -0.73) | -0.53 (-1.17, 0.12) | -1.84 (-2.46 ,-1.22) | -2.67 (-3.49, -1.86) | -2.82 (-3.77, -1.87) | -0.03 (-0.89, 0.83) |
| Pre-op score in 3 categories |  |  |  |  |  |  |
| lowest |  |  |  |  |  |  |
| middle | 7.03 (6.52, 7.54) | 5.69 (5.22, 6.17) | 6.74 (6.28 ,7.19) | 5.25 (4.60, 5.89) | 3.88 (3.13, 4.62) |  |
| highest | 11.79 (11.34, 12.25) | 11.96 (11.49, 12.43) | 11.08 (10.61 ,11.55) | 11.44 (10.77, 12.11) | 7.79 (7.09, 8.49) |  |
| Male (vs. female) | 2.06 (1.71, 2.40) | 1.60 (1.20, 1.99) | 2.63 (2.25 ,3.01) | 1.25 (0.75, 1.75) | 2.76 (2.17, 3.34) | 1.64 (1.12, 2.16) |
| Age |  |  |  |  |  |  |
| <50 |  |  |  |  |  |  |
| 50-69 | 0.58 (-0.90, 2.05) | 0.70 (-0.97, 2.36) | 0.93 (-0.65 ,2.50) | 1.37 (-0.74, 3.48) | 3.96 (1.49, 6.42) | 1.32 (-0.87, 3.51) |
| >70 | -0.07 (-1.55, 1.42) | -1.05 (-2.73, 0.63) | 0.78 (-0.81 ,2.37) | 1.43 (-0.70, 3.56) | 5.85 (3.37, 8.34) | 1.80 (-0.40, 4.00) |
| BMI |  |  |  |  |  |  |
| <25 |  |  |  |  |  |  |
| 25-30 | -0.83 (-1.29, -0.38) | -0.65 (-1.17, -0.14) | -1.13 (-1.62 ,-0.63) | -1.46 (-2.11, -0.80) | -1.33 (-2.10, -0.57) | -0.43 (-1.11, 0.25) |
| >30 | -2.18 (-2.66, -1.70) | -1.64 (-2.19, -1.10) | -2.73 (-3.26 ,-2.21) | -2.27 (-2.96, -1.58) | -2.42 (-3.23, -1.61) | -0.33 (-1.05, 0.39) |
| ASA |  |  |  |  |  |  |
| I |  |  |  |  |  |  |
| II | -1.61 (-2.14, -1.09) | -2.28 (-2.88, -1.69) | -1.51 (-2.07 ,-0.95) | -2.13 (-2.89, -1.37) | -2.22 (-3.10, -1.33) | -1.85 (-2.63, -1.06) |
| III-IV | -5.34 (-5.99, -4.69) | -6.74 (-7.48, -6.01) | -5.19 (-5.89 ,-4.50) | -4.50 (-5.42, -3.58) | -5.18 (-6.26, -4.10) | -4.57 (-5.53, -3.61) |
| Charnley |  |  |  |  |  |  |
| A |  |  |  |  |  |  |
| B1 | -0.08 (-0.47, 0.31) | -0.26 (-0.70, 0.18) | -0.24 (-0.66 ,0.18) | 0.02 (-0.54, 0.58) | 0.35 (-0.30, 1.00) | 0.02 (-0.56, 0.60) |
| B2 | 0.13 (-0.32, 0.58) | 0.35 (-0.16, 0.86) | -0.33 (-0.81 ,0.16) | 0.56 (-0.09, 1.21) | 0.89 (0.13, 1.65) | 1.95 (1.27, 2.62) |
| C | -1.21 (-2.14, -0.28) | -1.28 (-2.33, -0.23) | -0.71 (-1.70 ,0.28) | 0.48 (-0.86, 1.83) | 0.62 (-0.95, 2.19) | 1.51 (0.13, 2.88) |
| Hospital |  |  |  |  |  |  |
| Private Hospital |  |  |  |  |  |  |
| General Hospital | -2.31 (-2.89, -1.74) | -1.46 (-2.12, -0.81) | -2.02 (-2.65 ,-1.40) | -1.92 (-2.81, -1.04) | -1.92 (-2.95, -0.89) | 2.57 (1.68, 3.45) |
| University Medical Center | -4.30 (-5.90, -2.71) | -1.21 (-3.05, 0.63) | -3.37 (-5.12 ,-1.62) | -3.29 (-5.67, -0.90) | -2.19 (-4.98, 0.59) | 2.13 (-0.25, 4.51) |
| R-squared | 0.119 | 0.113 | 0.124 | 0.053 | 0.033 | 0.006 |

**Table 8: Demographics and outcomes of THA patients (EQ-5D-5L cohort)**

|  | **Q1 (least depr.)** | **Q2** | **Q3** | **Q4** | **Q5 (most depr.)** | **p-value** |
| --- | --- | --- | --- | --- | --- | --- |
| Total | 2628 | 2801 | 3050 | 3250 | 2659 |  |
| SES Z-score, median [range] | 1.3 [0.9, 2.8] | 0.6 [0.4, 0.9] | 0.2 [-0.1, 0.4] | -0.4 [-0.8, -0.1] | -1.37 [-6.13, -0.78] | |
| Demographics |  |  |  |  |  |  |
| Age, median [IQR] | 70.0 [63.0, 75.0] | 70.0 [63.0, 75.0] | 70.0 [63.0, 75.0] | 70.0 [64.0, 76.0] | 71.0 [63.5, 76.0] | <0.001 |
| <50 | 51 (2) | 80 (3) | 67 (2) | 52 (2) | 58 (2) | 0.012 |
| 50-69 | 1244 (47) | 1310 (47) | 1401 (46) | 1471 (45) | 1151 (43) |  |
| >70 | 1333 (51) | 1411 (50) | 1582 (52) | 1727 (53) | 1450 (55) |  |
| BMI, median [IQR] | 26.0 [23.7, 29.0] | 26.4 [24.0, 29.4] | 26.9 [24.2, 30.0] | 26.9 [24.2, 29.8] | 27.2 [24.7, 30.3] | <0.001 |
| <25 | 1143 (43) | 1126 (40) | 1099 (36) | 1172 (36) | 880 (33) | <0.001 |
| 25-30 | 1066 (41) | 1134 (40) | 1296 (42) | 1386 (43) | 1134 (43) |  |
| >30 | 419 (16) | 541 (19) | 655 (21) | 692 (21) | 645 (24) |  |
| Male | 1688 (64) | 1730 (62) | 1946 (64) | 2085 (64) | 1779 (67) | 0.044 |
| ASA |  |  |  |  |  | <0.001 |
| I | 494 (19) | 505 (18) | 561 (18) | 484 (15) | 332 (12) |  |
| II | 1687 (64) | 1760 (63) | 1876 (62) | 2022 (62) | 1712 (64) |  |
| III-IV | 447 (17) | 536 (19) | 613 (20) | 744 (23) | 615 (23) |  |
| Charnley |  |  |  |  |  | 0.03 |
| A | 1169 (44) | 1205 (43) | 1298 (43) | 1344 (41) | 1139 (43) |  |
| B1 | 849 (32) | 878 (31) | 973 (32) | 1064 (33) | 843 (32) |  |
| B2 | 562 (21) | 626 (22) | 677 (22) | 717 (22) | 598 (22) |  |
| C | 48 (2) | 92 (3) | 102 (3) | 125 (4) | 79 (3) |  |
| Smoking (yes) | 172 (7) | 212 (8) | 210 (7) | 252 (8) | 240 (9) | 0.001 |
| Previous surgery of the joint (yes) | 31 (1) | 26 (1) | 38 (1) | 28 (1) | 26 (1) | 0.564 |
| Year of surgery |  |  |  |  |  | NaN |
| 2014 | 0 (0) | 0 (0) | 0 (0) | 0 (0) | 0 (0) |  |
| 2015 | 0 (0) | 0 (0) | 0 (0) | 0 (0) | 0 (0) |  |
| 2016 | 0 (0) | 0 (0) | 0 (0) | 0 (0) | 0 (0) |  |
| 2017 | 0 (0) | 0 (0) | 0 (0) | 0 (0) | 0 (0) |  |
| 2018 | 0 (0) | 0 (0) | 0 (0) | 0 (0) | 0 (0) |  |
| 2019 | 1 (0) | 0 (0) | 0 (0) | 0 (0) | 0 (0) |  |
| 2020 | 232 (9) | 221 (8) | 212 (7) | 205 (6) | 191 (7) |  |
| 2021 | 1612 (61) | 1737 (62) | 1923 (63) | 1969 (61) | 1546 (58) |  |
| 2022 | 783 (30) | 843 (30) | 915 (30) | 1076 (33) | 922 (35) |  |
| Type of hospital |  |  |  |  |  | <0.001 |
| General Hospital | 1733 (66) | 2036 (73) | 2323 (76) | 2613 (80) | 2160 (81) |  |
| Private Hospital | 889 (34) | 758 (27) | 720 (24) | 620 (19) | 483 (18) |  |
| University Medical Center | 6 (0) | 7 (0) | 7 (0) | 17 (1) | 16 (1) |  |
| Fixation |  |  |  |  |  | <0.001 |
| Cemented | 322 (12) | 340 (12) | 499 (16) | 526 (16) | 602 (23) |  |
| Cementless | 2133 (81) | 2235 (80) | 2303 (76) | 2434 (75) | 1751 (66) |  |
| Hybrid | 173 (7) | 226 (8) | 248 (8) | 290 (9) | 306 (12) |  |
| Approach |  |  |  |  |  | <0.001 |
| Anterior | 1729 (66) | 1598 (57) | 1482 (49) | 1481 (46) | 1172 (44) |  |
| Anterolateral | 53 (2) | 70 (2) | 70 (2) | 60 (2) | 30 (1) |  |
| Other | 24 (1) | 30 (1) | 99 (3) | 60 (2) | 55 (2) |  |
| Posterolateral | 769 (29) | 1058 (38) | 1352 (44) | 1580 (49) | 1332 (50) |  |
| Straight lateral | 53 (2) | 45 (2) | 47 (2) | 69 (2) | 70 (3) |  |
| Contralateral procedure (yes) | 339 (13) | 399 (14) | 423 (14) | 453 (14) | 374 (14) | 0.23 |
| Outcomes |  |  |  |  |  |  |
| Preoperative outcomes, median [IQR] |  |  |  |  |  |  |
| EQ-5D-5L LSS | 60.0 [50.0, 70.0] | 60.0 [50.0, 70.0] | 60.0 [45.0, 70.0] | 60.0 [45.0, 70.0] | 55.0 [45.0, 65.0] |  |
| OHS | 50.0 [37.5, 62.5] | 47.9 [35.4, 60.4] | 47.9 [35.4, 60.4] | 47.9 [33.3, 60.4] | 43.8 [31.2, 58.3] |  |
| EQ VAS | 70.0 [51.0, 80.0] | 70.0 [51.0, 80.0] | 70.0 [50.0, 80.0] | 70.0 [50.0, 80.0] | 67.0 [50.0, 80.0] |  |
| NRS Pain in rest | 50.0 [30.0, 70.0] | 50.0 [30.0, 70.0] | 50.0 [30.0, 70.0] | 40.0 [30.0, 60.0] | 40.0 [20.0, 60.0] |  |
| NRS Pain during activity | 20.0 [20.0, 40.0] | 20.0 [10.0, 40.0] | 20.0 [10.0, 30.0] | 20.0 [10.0, 30.0] | 20.0 [10.0, 30.0] |  |
| 12-month follow-up outcomes, median [IQR] |  |  |  |  |  |  |
| EQ-5D-5L LSS | 95.0 [85.0, 100.0] | 95.0 [85.0, 100.0] | 95.0 [85.0, 100.0] | 95.0 [80.0, 100.0] | 90.0 [80.0, 100.0] |  |
| OHS | 93.8 [85.4, 100.0] | 93.8 [85.4, 100.0] | 93.8 [83.3, 97.9] | 93.8 [83.3, 97.9] | 91.7 [79.2, 97.9] |  |
| EQ VAS | 81.0 [71.0, 90.0] | 81.0 [71.0, 90.0] | 80.0 [70.0, 90.0] | 80.0 [70.0, 90.0] | 80.0 [70.0, 90.0] |  |
| NRS Pain in rest | 100.0 [90.0, 100.0] | 100.0 [90.0, 100.0] | 100.0 [90.0, 100.0] | 100.0 [90.0, 100.0] | 100.0 [90.0, 100.0] | |
| NRS Pain during activity | 100.0 [80.0, 100.0] | 100.0 [80.0, 100.0] | 100.0 [80.0, 100.0] | 100.0 [80.0, 100.0] | 100.0 [70.0, 100.0] | |
| Preoperative outcomes, n (%) ceiling |  |  |  |  |  |  |
| EQ-5D-5L LSS | 14 (0.5) | 5 (0.2) | 8 (0.3) | 10 (0.3) | 8 (0.3) |  |
| OHS | 2 (0.1) | 2 (0.1) | 1 (0.0) | 0 (0.0) | 1 (0.0) |  |
| EQ VAS | 47 (1.8) | 55 (2.0) | 64 (2.1) | 61 (1.9) | 36 (1.4) |  |
| NRS Pain in rest | 142 (5.4) | 153 (5.5) | 131 (4.3) | 148 (4.6) | 114 (4.3) |  |
| NRS Pain during activity | 18 (0.7) | 17 (0.6) | 13 (0.4) | 25 (0.8) | 17 (0.6) |  |
| 12-month follow-up outcomes, n (%) ceiling |  |  |  |  |  |  |
| EQ-5D-5L LSS | 1155 (43.9) | 1222 (43.6) | 1256 (41.2) | 1301 (40.0) | 995 (37.4) |  |
| OHS | 700 (27.1) | 745 (27.2) | 735 (24.6) | 721 (22.7) | 571 (22.0) |  |
| EQ VAS | 162 (6.2) | 206 (7.4) | 202 (6.7) | 220 (6.9) | 159 (6.1) |  |
| NRS Pain in rest | 1815 (69.2) | 1938 (69.4) | 2050 (67.5) | 2141 (66.0) | 1737 (65.5) |  |
| NRS Pain during activity | 1410 (53.8) | 1529 (54.8) | 1575 (51.8) | 1656 (51.1) | 1327 (50.1) |  |

**Table 9: Demographics and outcomes of TKA patients (EQ-5D-5L cohort)**

|  | **Q1 (least depr.)** | **Q2** | **Q3** | **Q4** | **Q5 (most depr.)** | **p-value** |
| --- | --- | --- | --- | --- | --- | --- |
| Total | 1312 | 1665 | 1971 | 2314 | 1929 |  |
| SES Z-score, median [range] | 1.2 [0.9, 2.8] | 0.6 [0.4, 0.9] | 0.2 [-0.1, 0.4] | -0.36 [-0.78, -0.07] | -1.4 [-7.8, -0.8] |  |
| Demographics |  |  |  |  |  |  |
| Age, median [IQR] | 70.0 [65.0, 75.0] | 70.0 [63.0, 75.0] | 69.0 [63.0, 75.0] | 70.0 [63.0, 75.0] | 70.0 [63.0, 75.0] | <0.001 |
| <50 | 9 (1) | 7 (0) | 15 (1) | 17 (1) | 20 (1) | 0.042 |
| 50-69 | 586 (45) | 815 (49) | 975 (49) | 1120 (48) | 937 (49) |  |
| >70 | 717 (55) | 843 (51) | 981 (50) | 1177 (51) | 972 (50) |  |
| BMI, median [IQR] | 27.8 [25.2, 31.1] | 28.3 [25.7, 31.6] | 28.7 [26.0, 32.2] | 29.0 [26.0, 32.3] | 29.3 [26.4, 33.0] | <0.001 |
| <25 | 351 (27) | 381 (23) | 405 (21) | 488 (21) | 345 (18) | <0.001 |
| 25-30 | 576 (44) | 724 (43) | 825 (42) | 950 (41) | 804 (42) |  |
| >30 | 385 (29) | 560 (34) | 741 (38) | 876 (38) | 780 (40) |  |
| Male | 811 (62) | 1027 (62) | 1187 (60) | 1370 (59) | 1219 (63) | 0.447 |
| ASA |  |  |  |  |  | 0.001 |
| I | 164 (12) | 208 (12) | 246 (12) | 258 (11) | 175 (9) |  |
| II | 850 (65) | 1092 (66) | 1251 (63) | 1530 (66) | 1237 (64) |  |
| III-IV | 298 (23) | 365 (22) | 474 (24) | 526 (23) | 517 (27) |  |
| Charnley |  |  |  |  |  | 0.014 |
| A | 541 (41) | 690 (41) | 781 (40) | 929 (40) | 805 (42) |  |
| B1 | 399 (30) | 561 (34) | 661 (34) | 802 (35) | 662 (34) |  |
| B2 | 319 (24) | 351 (21) | 457 (23) | 500 (22) | 407 (21) |  |
| C | 53 (4) | 63 (4) | 72 (4) | 83 (4) | 55 (3) |  |
| Smoking (yes) | 77 (6) | 85 (5) | 112 (6) | 160 (7) | 158 (8) | 0.015 |
| Previous surgery of the joint (yes) | 270 (21) | 391 (23) | 510 (26) | 566 (24) | 482 (25) | 0.004 |
| Year of surgery |  |  |  |  |  | NaN |
| 2014 | 0 (0) | 0 (0) | 0 (0) | 0 (0) | 0 (0) |  |
| 2015 | 0 (0) | 0 (0) | 0 (0) | 0 (0) | 0 (0) |  |
| 2016 | 0 (0) | 0 (0) | 0 (0) | 0 (0) | 0 (0) |  |
| 2017 | 0 (0) | 0 (0) | 0 (0) | 0 (0) | 0 (0) |  |
| 2018 | 1 (0) | 0 (0) | 0 (0) | 0 (0) | 0 (0) |  |
| 2019 | 0 (0) | 2 (0) | 0 (0) | 0 (0) | 0 (0) |  |
| 2020 | 108 (8) | 126 (8) | 148 (8) | 149 (6) | 108 (6) |  |
| 2021 | 829 (63) | 1020 (61) | 1259 (64) | 1455 (63) | 1199 (62) |  |
| 2022 | 374 (29) | 517 (31) | 564 (29) | 710 (31) | 622 (32) |  |
| Type of hospital |  |  |  |  |  | 0.033 |
| General Hospital | 1035 (79) | 1333 (80) | 1551 (79) | 1879 (81) | 1567 (81) |  |
| Private Hospital | 274 (21) | 324 (19) | 412 (21) | 424 (18) | 349 (18) |  |
| University Medical Center | 3 (0) | 8 (0) | 8 (0) | 11 (0) | 13 (1) |  |
| Fixation |  |  |  |  |  | 0.002 |
| Cemented | 1214 (93) | 1543 (93) | 1816 (92) | 2107 (91) | 1712 (89) |  |
| Cementless | 62 (5) | 89 (5) | 118 (6) | 164 (7) | 142 (7) |  |
| Hybrid | 36 (3) | 33 (2) | 37 (2) | 43 (2) | 75 (4) |  |
| Approach |  |  |  |  |  | 0.184 |
| Lateral parapatellar | 8 (1) | 6 (0) | 17 (1) | 13 (1) | 18 (1) |  |
| Medial parapatellar | 1273 (97) | 1627 (98) | 1906 (97) | 2253 (97) | 1881 (98) |  |
| Other | 6 (0) | 4 (0) | 4 (0) | 6 (0) | 3 (0) |  |
| Vastus (mid/sub) | 25 (2) | 28 (2) | 44 (2) | 42 (2) | 27 (1) |  |
| Contralateral procedure (yes) | 225 (17) | 245 (15) | 338 (17) | 360 (16) | 291 (15) | 0.127 |
| Outcomes |  |  |  |  |  |  |
| Preoperative outcomes, median [IQR] |  |  |  |  |  |  |
| EQ-5D-5L LSS | 65.0 [55.0, 70.0] | 65.0 [50.0, 70.0] | 65.0 [55.0, 70.0] | 65.0 [55.0, 70.0] | 60.0 [50.0, 70.0] |  |
| OHS | 50.0 [39.6, 60.4] | 47.9 [39.6, 60.4] | 47.9 [37.5, 60.4] | 47.9 [37.5, 58.3] | 45.8 [33.3, 56.2] |  |
| EQ VAS | 70.0 [58.0, 80.0] | 70.0 [59.0, 81.0] | 70.0 [60.0, 80.0] | 70.0 [59.0, 81.0] | 70.0 [55.0, 80.0] |  |
| NRS Pain in rest | 50.0 [30.0, 70.0] | 50.0 [30.0, 70.0] | 50.0 [30.0, 70.0] | 40.0 [30.0, 70.0] | 40.0 [30.0, 60.0] |  |
| NRS Pain during activity | 20.0 [10.0, 40.0] | 20.0 [20.0, 40.0] | 20.0 [10.0, 30.0] | 20.0 [10.0, 30.0] | 20.0 [10.0, 30.0] |  |
| 12-month follow-up outcomes, median [IQR] |  |  |  |  |  |  |
| EQ-5D-5L LSS | 90.0 [80.0, 100.0] | 90.0 [80.0, 100.0] | 90.0 [80.0, 100.0] | 90.0 [80.0, 100.0] | 90.0 [80.0, 100.0] |  |
| OHS | 87.5 [75.0, 93.8] | 87.5 [75.0, 93.8] | 87.5 [75.0, 93.8] | 85.4 [70.8, 93.8] | 85.4 [68.8, 93.8] |  |
| EQ VAS | 80.0 [70.0, 90.0] | 80.0 [70.0, 90.0] | 80.0 [70.0, 90.0] | 80.0 [70.0, 90.0] | 80.0 [70.0, 90.0] |  |
| NRS Pain in rest | 100.0 [80.0, 100.0] | 100.0 [80.0, 100.0] | 90.0 [80.0, 100.0] | 90.0 [80.0, 100.0] | 90.0 [70.0, 100.0] |  |
| NRS Pain during activity | 90.0 [60.0, 100.0] | 90.0 [70.0, 100.0] | 80.0 [60.0, 100.0] | 80.0 [60.0, 100.0] | 80.0 [60.0, 100.0] |  |
| NRS Satisfaction | 80.0 [70.0, 90.0] | 80.0 [70.0, 90.0] | 80.0 [70.0, 90.0] | 80.0 [70.0, 90.0] | 80.0 [70.0, 90.0] |  |
| Preoperative outcomes, n (%) ceiling |  |  |  |  |  |  |
| EQ-5D-5L LSS | 3 (0.2) | 9 (0.5) | 3 (0.2) | 10 (0.4) | 12 (0.6) |  |
| OHS | 0 (0.0) | 0 (0.0) | 0 (0.0) | 0 (0.0) | 1 (0.1) |  |
| EQ VAS | 24 (1.8) | 34 (2.0) | 34 (1.7) | 51 (2.2) | 52 (2.7) |  |
| NRS Pain in rest | 96 (7.4) | 103 (6.2) | 104 (5.3) | 99 (4.3) | 95 (5.0) |  |
| NRS Pain during activity | 5 (0.4) | 13 (0.8) | 6 (0.3) | 10 (0.4) | 12 (0.6) |  |
| 12-month follow-up outcomes, n (%) ceiling |  |  |  |  |  |  |
| EQ-5D-5L LSS | 407 (31.0) | 523 (31.4) | 561 (28.5) | 659 (28.5) | 559 (29.0) |  |
| OHS | 78 (6.1) | 101 (6.3) | 111 (5.9) | 121 (5.4) | 101 (5.4) |  |
| EQ VAS | 68 (5.2) | 77 (4.7) | 84 (4.3) | 103 (4.5) | 104 (5.5) |  |
| NRS Pain in rest | 690 (52.8) | 868 (52.6) | 977 (49.6) | 1114 (48.4) | 945 (49.2) |  |
| NRS Pain during activity | 439 (33.7) | 544 (32.9) | 605 (30.8) | 667 (29.0) | 620 (32.3) |  |
| NRS Satisfaction | 294 (23.5) | 399 (24.6) | 434 (22.3) | 510 (22.5) | 467 (24.6) |  |

**Table 10: Association between socioeconomic status and preoperative health status of THA patients (EQ-5D-5L cohort)**

|  | **LSS** | **VAS** | **OHS** | **NRS Pain in rest** | **NRS Pain during activity** |
| --- | --- | --- | --- | --- | --- |
| **Variables** | **Coefficient (95% CI)** | **Coefficient (95% CI)** | **Coefficient (95% CI)** | **Coefficient (95% CI)** | **Coefficient (95% CI)** |
| Intercept | 61.34 (59.49, 63.19) | 64.68 (62.16, 67.19) | 50.16 (48.53 ,51.80) | 51.78 (48.71, 54.86) | 29.42 (27.09, 31.75) |
| SES |  |  |  |  |  |
| Q1, least deprived |  |  |  |  |  |
| Q2 | -1.19 (-1.98, -0.39) | 0.95 (-0.13, 2.03) | -0.74 (-1.33 ,-0.14) | -0.15 (-1.47, 1.16) | -1.19 (-2.19, -0.19) |
| Q3 | -2.13 (-2.91, -1.35) | -0.04 (-1.10, 1.02) | -1.50 (-2.06 ,-0.93) | -1.29 (-2.59, 0.00) | -1.47 (-2.45, -0.49) |
| Q4 | -1.78 (-2.55, -1.01) | -0.06 (-1.11, 0.98) | -1.77 (-2.32 ,-1.22) | -2.11 (-3.39, -0.83) | -2.26 (-3.23, -1.29) |
| Q5, most deprived | -2.32 (-3.13, -1.52) | -1.50 (-2.60, -0.40) | -3.03 (-3.60 ,-2.45) | -4.18 (-5.53, -2.84) | -2.56 (-3.58, -1.54) |
| Male (vs. female) | 3.07 (2.55, 3.58) | 3.64 (2.94, 4.34) | 6.48 (6.13 ,6.83) | 4.74 (3.88, 5.59) | 3.86 (3.21, 4.50) |
| Age |  |  |  |  |  |
| <50 |  |  |  |  |  |
| 50-69 | 4.20 (2.50, 5.90) | 3.87 (1.55, 6.19) | 3.76 (2.29 ,5.23) | 4.31 (1.48, 7.14) | 3.52 (1.37, 5.66) |
| >70 | 4.60 (2.89, 6.32) | 6.18 (3.84, 8.51) | 4.86 (3.38 ,6.34) | 6.94 (4.09, 9.79) | 4.73 (2.57, 6.89) |
| BMI |  |  |  |  |  |
| <25 |  |  |  |  |  |
| 25-30 | -1.77 (-2.32, -1.22) | -1.26 (-2.01, -0.50) | -1.78 (-2.23 ,-1.32) | -1.73 (-2.65, -0.81) | -2.25 (-2.95, -1.56) |
| >30 | -5.17 (-5.87, -4.48) | -4.11 (-5.06, -3.16) | -5.26 (-5.75 ,-4.78) | -5.16 (-6.32, -4.00) | -5.44 (-6.32, -4.56) |
| ASA |  |  |  |  |  |
| I |  |  |  |  |  |
| II | -2.65 (-3.35, -1.95) | -3.73 (-4.69, -2.78) | -2.59 (-3.11 ,-2.07) | -2.32 (-3.49, -1.15) | -1.69 (-2.58, -0.81) |
| III-IV | -6.65 (-7.54, -5.76) | -9.31 (-10.53, -8.09) | -7.10 (-7.74 ,-6.46) | -4.95 (-6.44, -3.47) | -4.65 (-5.78, -3.53) |
| Charnley |  |  |  |  |  |
| A |  |  |  |  |  |
| B1 | 0.12 (-0.45, 0.69) | 0.35 (-0.43, 1.12) | -0.23 (-0.62 ,0.16) | 0.80 (-0.14, 1.75) | 0.41 (-0.30, 1.13) |
| B2 | 1.66 (1.02, 2.30) | 0.40 (-0.47, 1.27) | 1.45 (1.00 ,1.90) | 0.31 (-0.76, 1.37) | 1.74 (0.94, 2.55) |
| C | -2.58 (-4.01, -1.14) | -1.06 (-3.02, 0.91) | -3.19 (-4.10 ,-2.27) | -3.97 (-6.36, -1.58) | -1.32 (-3.13, 0.49) |
| Hospital |  |  |  |  |  |
| Private Hospital |  |  |  |  |  |
| General Hospital | -3.51 (-4.12, -2.91) | -0.73 (-1.55, 0.10) | -0.47 (-1.05 ,0.11) | -7.75 (-8.76, -6.74) | -3.99 (-4.75, -3.22) |
| University Medical Center | -0.34 (-4.39, 3.71) | 3.56 (-1.94, 9.06) | -7.45 (-9.06 ,-5.83) | -6.84 (-13.77, 0.08) | -4.98 (-10.28, 0.31) |
| R-squared | 0.074 | 0.039 | 0.105 | 0.049 | 0.046 |

**Table 11: Association between socioeconomic status and preoperative health status of TKA patients (EQ-5D-5L cohort)**

|  | **LSS** | **VAS** | **OKS** | **NRS Pain in rest** | **NRS Pain during activity** |
| --- | --- | --- | --- | --- | --- |
| **Variables** | **Coefficient (95% CI)** | **Coefficient (95% CI)** | **Coefficient (95% CI)** | **Coefficient (95% CI)** | **Coefficient (95% CI)** |
| Intercept | 64.81 (61.30, 68.33) | 67.20 (62.52, 71.88) | 52.68 (48.78 ,56.58) | 47.30 (40.99, 53.62) | 29.05 (24.33, 33.77) |
| SES |  |  |  |  |  |
| Q1, least deprived |  |  |  |  |  |
| Q2 | -0.66 (-1.67, 0.35) | 0.70 (-0.65, 2.05) | 0.16 (-0.95 ,1.27) | 0.72 (-1.10, 2.54) | -0.06 (-1.42, 1.30) |
| Q3 | -0.53 (-1.50, 0.45) | 0.83 (-0.47, 2.13) | -0.16 (-1.23 ,0.90) | -1.70 (-3.46, 0.06) | -1.64 (-2.95, -0.33) |
| Q4 | -0.60 (-1.54, 0.35) | 0.66 (-0.60, 1.92) | -0.58 (-1.61 ,0.46) | -2.77 (-4.48, -1.06) | -1.41 (-2.69, -0.14) |
| Q5, most deprived | -0.78 (-1.76, 0.20) | 0.63 (-0.68, 1.95) | -2.50 (-3.57 ,-1.42) | -4.90 (-6.66, -3.13) | -1.97 (-3.29, -0.65) |
| Male (vs. female) | 2.74 (2.15, 3.33) | 2.88 (2.09, 3.67) | 6.10 (5.45 ,6.75) | 5.07 (4.00, 6.14) | 3.55 (2.76, 4.35) |
| Age |  |  |  |  |  |
| <50 |  |  |  |  |  |
| 50-69 | 3.19 (-0.15, 6.52) | 5.29 (0.84, 9.74) | 1.99 (-1.72 ,5.70) | 8.16 (2.16, 14.17) | 2.08 (-2.40, 6.57) |
| >70 | 3.87 (0.52, 7.22) | 7.13 (2.66, 11.59) | 2.40 (-1.33 ,6.12) | 12.08 (6.05, 18.10) | 4.69 (0.19, 9.19) |
| BMI |  |  |  |  |  |
| <25 |  |  |  |  |  |
| 25-30 | -1.80 (-2.56, -1.04) | -2.02 (-3.04, -1.01) | -3.13 (-3.96 ,-2.29) | -3.09 (-4.46, -1.72) | -2.51 (-3.53, -1.49) |
| >30 | -3.64 (-4.46, -2.82) | -3.53 (-4.62, -2.43) | -5.89 (-6.79 ,-4.99) | -4.69 (-6.16, -3.22) | -3.96 (-5.06, -2.86) |
| ASA |  |  |  |  |  |
| I |  |  |  |  |  |
| II | -1.99 (-2.94, -1.05) | -4.25 (-5.52, -2.99) | -2.77 (-3.81 ,-1.73) | -1.66 (-3.37, 0.05) | -1.39 (-2.67, -0.12) |
| III-IV | -5.55 (-6.68, -4.43) | -9.85 (-11.35, -8.34) | -6.79 (-8.02 ,-5.55) | -4.06 (-6.09, -2.03) | -3.07 (-4.59, -1.55) |
| Charnley |  |  |  |  |  |
| A |  |  |  |  |  |
| B1 | -0.54 (-1.21, 0.13) | 0.30 (-0.59, 1.19) | -0.29 (-1.02 ,0.44) | 0.86 (-0.34, 2.07) | 0.35 (-0.55, 1.25) |
| B2 | 1.18 (0.42, 1.94) | 1.17 (0.15, 2.18) | 1.37 (0.54 ,2.21) | 0.95 (-0.42, 2.32) | 0.84 (-0.18, 1.86) |
| C | -1.59 (-3.18, -0.01) | -0.10 (-2.23, 2.02) | -0.67 (-2.41 ,1.06) | 1.06 (-1.80, 3.92) | 1.74 (-0.40, 3.88) |
| Hospital |  |  |  |  |  |
| Private Hospital |  |  |  |  |  |
| General Hospital | -2.39 (-3.16, -1.62) | 0.24 (-0.78, 1.27) | -1.93 (-2.77 ,-1.08) | -5.55 (-6.93, -4.17) | -3.01 (-4.04, -1.97) |
| University Medical Center | 1.08 (-3.14, 5.30) | 4.11 (-1.58, 9.79) | -8.95 (-13.61 ,-4.29) | -0.35 (-8.21, 7.52) | -1.87 (-7.67, 3.94) |
| R-squared | 0.051 | 0.039 | 0.098 | 0.041 | 0.031 |

**Table 12: Association between socioeconomic status and 12-month follow-up health status of THA patients (EQ-5D-5L cohort)**

|  | **LSS** | **VAS** | **OHS** | **NRS Pain in rest** | **NRS Pain during activity** |
| --- | --- | --- | --- | --- | --- |
| **Variables** | **Coefficient (95% CI)** | **Coefficient (95% CI)** | **Coefficient (95% CI)** | **Coefficient (95% CI)** | **Coefficient (95% CI)** |
| Intercept | 90.22 (88.49, 91.95) | 80.38 (78.17, 82.58) | 92.15 (90.28 ,94.02) | 88.59 (86.29, 90.89) | 85.58 (82.76, 88.40) |
| SES |  |  |  |  |  |
| Q1, least deprived |  |  |  |  |  |
| Q2 | 0.54 (-0.18, 1.26) | 0.54 (-0.39, 1.46) | 0.14 (-0.64 ,0.93) | 0.46 (-0.49, 1.41) | 0.15 (-1.03, 1.32) |
| Q3 | 0.24 (-0.47, 0.94) | 0.41 (-0.50, 1.32) | -0.24 (-1.01 ,0.53) | 0.10 (-0.83, 1.03) | -0.13 (-1.29, 1.03) |
| Q4 | 0.16 (-0.54, 0.86) | -0.17 (-1.07, 0.73) | -0.45 (-1.21 ,0.32) | 0.31 (-0.61, 1.23) | 0.15 (-1.00, 1.29) |
| Q5, most deprived | -0.47 (-1.21, 0.26) | 0.03 (-0.92, 0.97) | -1.16 (-1.97 ,-0.36) | -0.31 (-1.28, 0.66) | -0.97 (-2.17, 0.24) |
| Pre-op score in 3 categories |  |  |  |  |  |
| lowest |  |  |  |  |  |
| middle | 4.63 (4.05, 5.21) | 3.91 (3.21, 4.61) | 3.26 (2.66 ,3.86) | 3.88 (3.10, 4.66) | 1.68 (0.73, 2.64) |
| highest | 7.61 (7.03, 8.18) | 9.41 (8.69, 10.12) | 5.83 (5.22 ,6.44) | 7.84 (7.02, 8.66) | 4.44 (3.53, 5.35) |
| Male (vs. female) | 1.44 (0.97, 1.90) | 1.25 (0.65, 1.85) | 1.69 (1.18 ,2.20) | 1.25 (0.63, 1.86) | 2.08 (1.31, 2.84) |
| Age |  |  |  |  |  |
| <50 |  |  |  |  |  |
| 50-69 | 0.15 (-1.40, 1.69) | -0.07 (-2.06, 1.92) | 0.04 (-1.64 ,1.72) | 1.70 (-0.34, 3.74) | 2.41 (-0.12, 4.94) |
| >70 | -1.28 (-2.84, 0.28) | -1.76 (-3.77, 0.24) | -1.45 (-3.15 ,0.24) | 1.00 (-1.05, 3.06) | 3.24 (0.69, 5.79) |
| BMI |  |  |  |  |  |
| <25 |  |  |  |  |  |
| 25-30 | -1.43 (-1.93, -0.92) | -1.50 (-2.15, -0.85) | -1.51 (-2.06 ,-0.96) | -1.25 (-1.91, -0.58) | -1.71 (-2.53, -0.88) |
| >30 | -3.28 (-3.91, -2.64) | -3.19 (-4.00, -2.37) | -3.40 (-4.10 ,-2.70) | -2.04 (-2.88, -1.21) | -2.96 (-4.00, -1.92) |
| ASA |  |  |  |  |  |
| I |  |  |  |  |  |
| II | -1.14 (-1.78, -0.50) | -2.67 (-3.49, -1.85) | -1.32 (-2.02 ,-0.63) | -0.84 (-1.69, 0.00) | -1.28 (-2.33, -0.24) |
| III-IV | -3.69 (-4.50, -2.87) | -7.21 (-8.26, -6.16) | -3.81 (-4.70 ,-2.92) | -1.93 (-3.01, -0.86) | -2.32 (-3.65, -0.99) |
| Charnley |  |  |  |  |  |
| A |  |  |  |  |  |
| B1 | -0.95 (-1.47, -0.44) | -0.85 (-1.52, -0.18) | -1.44 (-2.01 ,-0.88) | -0.53 (-1.21, 0.15) | -0.64 (-1.48, 0.21) |
| B2 | -1.06 (-1.64, -0.48) | -0.72 (-1.46, 0.03) | -0.96 (-1.60 ,-0.33) | 0.40 (-0.36, 1.17) | 0.37 (-0.58, 1.32) |
| C | -3.33 (-4.63, -2.02) | -3.20 (-4.89, -1.51) | -2.83 (-4.26 ,-1.40) | -1.13 (-2.85, 0.60) | -3.11 (-5.25, -0.97) |
| Hospital |  |  |  |  |  |
| Private Hospital |  |  |  |  |  |
| General Hospital | -3.28 (-3.83, -2.73) | -2.17 (-2.88, -1.46) | -3.80 (-4.40 ,-3.20) | -2.83 (-3.56, -2.10) | -4.46 (-5.36, -3.56) |
| University Medical Center | -6.32 (-10.00, -2.64) | -5.90 (-10.61, -1.20) | -5.09 (-9.29 ,-0.88) | -1.82 (-6.80, 3.16) | -5.67 (-11.91, 0.56) |
| R-squared | 0.106 | 0.094 | 0.086 | 0.043 | 0.027 |

**Table 13: Association between socioeconomic status and 12-month follow-up health status of TKA patients (EQ-5D-5L cohort)**

|  | **LSS** | **VAS** | **OKS** | **NRS Pain in rest** | **NRS Pain during activity** | **NRS Satisfaction** |
| --- | --- | --- | --- | --- | --- | --- |
| **Variables** | **Coefficient (95% CI)** | **Coefficient (95% CI)** | **Coefficient (95% CI)** | **Coefficient (95% CI)** | **Coefficient (95% CI)** | **Coefficient (95% CI)** |
| Intercept | 86.84 (83.27, 90.40) | 81.01 (76.61, 85.42) | 81.68 (77.57 ,85.78) | 84.98 (79.56, 90.39) | 74.87 (68.59, 81.15) | 82.42 (77.31, 87.53) |
| SES |  |  |  |  |  |  |
| Q1, least deprived |  |  |  |  |  |  |
| Q2 | -0.32 (-1.33, 0.69) | -0.28 (-1.54, 0.98) | -0.12 (-1.28 ,1.03) | -0.59 (-2.14, 0.95) | -0.50 (-2.30, 1.30) | 0.03 (-1.46, 1.53) |
| Q3 | -1.02 (-1.99, -0.04) | -0.96 (-2.18, 0.26) | -0.25 (-1.36 ,0.87) | -1.12 (-2.61, 0.37) | -1.39 (-3.13, 0.35) | -0.06 (-1.50, 1.38) |
| Q4 | -1.38 (-2.33, -0.44) | -1.19 (-2.37, 0.00) | -1.06 (-2.14 ,0.02) | -1.60 (-3.04, -0.15) | -2.24 (-3.92, -0.55) | -0.22 (-1.62, 1.18) |
| Q5, most deprived | -1.72 (-2.71, -0.74) | -0.71 (-1.95, 0.52) | -1.91 (-3.03 ,-0.79) | -1.81 (-3.31, -0.30) | -1.92 (-3.67, -0.17) | 0.04 (-1.41, 1.48) |
| Pre-op score in 3 categories |  |  |  |  |  |  |
| lowest |  |  |  |  |  |  |
| middle | 6.20 (5.45, 6.95) | 4.80 (3.90, 5.70) | 5.56 (4.75 ,6.38) | 5.56 (4.37, 6.74) | 2.35 (1.02, 3.69) |  |
| highest | 9.46 (8.71, 10.22) | 10.78 (9.90, 11.67) | 9.80 (8.96 ,10.65) | 12.51 (11.28, 13.75) | 7.99 (6.71, 9.26) |  |
| Male (vs. female) | 1.36 (0.77, 1.96) | 1.17 (0.43, 1.92) | 2.90 (2.22 ,3.59) | 1.25 (0.34, 2.16) | 3.13 (2.07, 4.19) | 2.19 (1.32, 3.06) |
| Age |  |  |  |  |  |  |
| <50 |  |  |  |  |  |  |
| 50-69 | -0.85 (-4.19, 2.50) | -1.91 (-6.08, 2.26) | -0.06 (-3.94 ,3.82) | -1.48 (-6.58, 3.61) | 3.19 (-2.74, 9.12) | 1.51 (-3.35, 6.36) |
| >70 | -1.03 (-4.38, 2.32) | -2.89 (-7.07, 1.29) | -1.03 (-4.91 ,2.86) | -1.87 (-6.98, 3.25) | 4.28 (-1.67, 10.23) | 0.33 (-4.53, 5.20) |
| BMI |  |  |  |  |  |  |
| <25 |  |  |  |  |  |  |
| 25-30 | -0.19 (-0.95, 0.57) | -0.69 (-1.64, 0.27) | -0.61 (-1.48 ,0.26) | 0.00 (-1.16, 1.17) | 0.01 (-1.35, 1.37) | -0.64 (-1.76, 0.47) |
| >30 | -0.93 (-1.75, -0.11) | -1.50 (-2.53, -0.47) | -2.06 (-3.00 ,-1.11) | -0.47 (-1.72, 0.79) | -0.92 (-2.38, 0.54) | -0.55 (-1.75, 0.65) |
| ASA |  |  |  |  |  |  |
| I |  |  |  |  |  |  |
| II | -1.82 (-2.77, -0.87) | -3.51 (-4.70, -2.32) | -1.55 (-2.64 ,-0.47) | -1.78 (-3.23, -0.33) | -2.09 (-3.78, -0.40) | -1.99 (-3.38, -0.60) |
| III-IV | -4.58 (-5.71, -3.45) | -7.95 (-9.37, -6.53) | -4.44 (-5.74 ,-3.15) | -4.12 (-5.84, -2.40) | -4.16 (-6.17, -2.15) | -3.34 (-4.99, -1.69) |
| Charnley |  |  |  |  |  |  |
| A |  |  |  |  |  |  |
| B1 | 0.48 (-0.19, 1.14) | -0.97 (-1.81, -0.13) | 0.07 (-0.69 ,0.84) | 0.39 (-0.63, 1.41) | 0.64 (-0.55, 1.83) | 0.51 (-0.47, 1.49) |
| B2 | 1.09 (0.33, 1.85) | -0.11 (-1.06, 0.84) | 0.28 (-0.59 ,1.15) | 0.91 (-0.25, 2.08) | 1.11 (-0.25, 2.46) | 2.43 (1.32, 3.55) |
| C | -2.12 (-3.71, -0.54) | -2.25 (-4.25, -0.26) | -2.00 (-3.82 ,-0.19) | 0.09 (-2.33, 2.52) | -0.29 (-3.12, 2.54) | 0.44 (-1.89, 2.77) |
| Hospital |  |  |  |  |  |  |
| Private Hospital |  |  |  |  |  |  |
| General Hospital | -3.06 (-3.83, -2.29) | -2.42 (-3.38, -1.46) | -3.02 (-3.89 ,-2.15) | -3.67 (-4.84, -2.49) | -4.75 (-6.11, -3.38) | -2.97 (-4.09, -1.85) |
| University Medical Center | -6.53 (-10.75, -2.30) | -6.95 (-12.34, -1.57) | -7.95 (-12.80 ,-3.09) | -4.14 (-10.88, 2.60) | -8.06 (-15.82, -0.30) | -7.34 (-13.61, -1.07) |
| R-squared | 0.104 | 0.104 | 0.116 | 0.063 | 0.039 | 0.012 |

**Table 14: Percentage of inequality explained by each EQ-5D-5L and OHS/OKS dimension (EQ-5D-5L cohort)**

|  | THA | | TKA | |
| --- | --- | --- | --- | --- |
|  | Preoperative | 12-month follow-up | Preoperative | 12-month follow-up |
| EQ-5D-5L |  |  |  |  |
| Mobility | 15 | 23 | 8 | 25 |
| Self-care | 21 | 53 | 29 | 10 |
| Usual activities | 31 | 51 | 45 | 25 |
| Pain/discomfort | 19 | 62 | 13 | 22 |
| Anxiety/depression | 13 | 61 | 5 | 16 |
| OHS/OKS |  |  |  |  |
| Function | 57 | 47 | 53 | 54 |
| Pain | 43 | 46 | 50 | 41 |
